# OhsR acts as an organic peroxide-sensing transcriptional activator using an *S*-mycothiolation mechanism in *Corynebacterium glutamicum*

**DOI:** 10.1186/s12934-018-1048-y

**Published:** 2018-12-26

**Authors:** Meiru Si, Tao Su, Can Chen, Jinfeng Liu, Zhijin Gong, Chengchuan Che, GuiZhi Li, Ge Yang

**Affiliations:** 1 College of Life Sciences, Qufu Normal University, Qufu, 273165 Shandong China; 2 College of Life Science and Agronomy, Zhoukou Normal University, Zhoukou, 466001 Henan China

**Keywords:** MarR, Organic peroxide stress, Transcription regulation, *C. glutamicum*, *S*-mycothiolation

## Abstract

**Background:**

*Corynebacterium glutamicum* is a well-known producer of various l-amino acids in industry. During the fermenting process, *C. glutamicum* unavoidably encounters oxidative stress due to a specific reactive oxygen species (ROS) produced by consistent adverse conditions. To combat the ROS, *C. glutamicum* has developed many common disulfide bond-based regulatory devices to control a specific set of antioxidant genes. However, nothing is known about the mixed disulfide between the protein thiol groups and the mycothiol (MSH) (*S*-mycothiolation)-based sensor. In addition, no OhrR (organic hydroperoxide resistance regulator) homologs and none of the organic hydroperoxide reductase (Ohr) sensors have been described in the alkyl hydroperoxide reductase CF-missing *C. glutamicum,* while organic hydroperoxides (OHPs)-specific Ohr was a core detoxification system.

**Results:**

In this study, we showed that the *C. glutamicum* OhsR acted as an OHPs sensor that activated *ohr* expression. OhsR conferred resistance to cumene hydroperoxide (CHP) and *t*-butyl hydroperoxide but not H_2_O_2_, hypochlorous acid, and diamide; this outcome was substantiated by the fact that the *ohsR*-deficient mutant was sensitive to OHPs but not inorganic peroxides. The DNA binding activity of OhsR was specifically activated by CHP. Mutational analysis of the two cysteines (Cys125 and Cys261) showed that Cys125 was primarily responsible for the activation of DNA binding. The oxidation of Cys125 produced a sulfenic acid (C125-SOH) that subsequently reacted with MSH to generate *S*-mycothiolation that was required to activate the *ohr* expression. Therefore, OhsR regulated the ohr expression using an *S*-mycothiolation mechanism in vivo.

**Conclusion:**

This is the first report demonstrating that the regulatory OhsR specifically sensed OHPs stress and responded to it by activating a specific *ohr* gene under its control using an *S*-mycothiolated mechanism.

**Electronic supplementary material:**

The online version of this article (10.1186/s12934-018-1048-y) contains supplementary material, which is available to authorized users.

## Background

During normal cellular functions or under unfavorable external environmental stimuli, bacteria unavoidably produce significant levels of reactive oxygen species (ROS), including hydrogen peroxide (H_2_O_2_), superoxide radical (O_2_^·−^), and organic peroxides (OHPs) [1]. The accumulation of excessive concentrations of ROS is toxic to biological systems, leading to DNA damage, lipid peroxidation, protein oxidation, and eventually causing various diseases, such as cancer, vascular and neurodegenerative disorders, and aging [2]. Among the external environmental stimuli, OHPs cause the most serious damage to the cells due to their ability to generate reactive organic radicals through the reaction with membranes, free fatty acids, or other macromolecules [3]. Therefore, OHPs detoxification is critical for bacterial survival. Bacteria have evolved diverse defense strategies against OHPs toxicity, including enzymes and low molecular weight antioxidants [4–11]. The OHPs detoxification enzymes alkyl hydroperoxide reductase subunit CF (AhpCF) and organic hydroperoxide resistance (Ohr) are the two core detoxification systems in bacteria [6, 7]. They can reduce OHPs into their corresponding alcohols. In addition to enzymes, another unique feature of the antioxidant system is the presence of low molecular weight thiols, such as tripeptide glutathione (GSH; γ-l-glutamyl-l-cysteinylglycine), 1-d-myo-inosityl 2-(*N*-acetyl-l-cysteinyl) amido-2-deoxy-d-glucopyranoside (mycothiol or MSH) and bacillithiol (BSH) [6, 7, 11, 12]. The low molecular weight thiols have a role in OHPs detoxification in vivo, confirmed by the increased sensitivity to cumene hydroperoxide (CHP), menadione (MD), and tert-butyl hydroperoxide (*t*-BHP) in MSH-deficient *Mycobacterium smegmatis*, *M. tuberculosis* and *Corynebacterium glutamicum* mutant strains [8, 9, 11].

Several studies have demonstrated that the diverse defense systems against oxidant toxicity are coordinated by thiol-based redox transcription sensors that sense exclusive ROS, modulate the appropriate regulon transcription, and finally confer resistance to oxidative stress and restore the redox balance [13–15]. Direct sensing and the rapid responses of thiol-based transcription factors are considered to be an efficient way to enhance the survival of the organism under oxidation stress. Thiol-based redox regulators control the antioxidant gene expression by versatile posttranslational thiol-modifications mechanisms, including the disulfides-switch model (sulfenic acid, disulfide bond, and *S*-thiolation), Cys-phosphorylation and Cys-alkylation. Although many of the common disulfide bond model-based regulators in bacteria have been well described, and *S*-thiolations in eukaryotes have emerged as the major redox-regulatory mechanism of redox sensing transcription factors, the *S*-thiolation-based regulation model (*S*-glutathionylation, *S*-mycothiolation and *S*-bacillithiolation) in bacteria has so far only been shown for a few selected sensors, such as the *Escherichia coli* OxyR (the thiol-based redox sensor for peroxides) and *B. subtilis* OhrR (organic hydroperoxide resistance regulator) [16, 17]. In addition, researchers found that each of the many regulators sensed a specific oxidant and responded to it by activating or depressing a specific set of genes under its control. For example, in bacteria, OxyR and OhrR respond to peroxide and organic hydroperoxide stress, respectively, while SoxRS (a sensor of superoxide and nitric-oxide stress) specifically responds to superoxide stress [4, 18, 19]. However, the exact molecular mechanism in different stimulus-segregating regulators or specific stimulus-sensing regulators remains to be elucidated to a large degree.

*Corynebacterium glutamicum*, a well-known various l-amino acid producer in industry and a model organism for systems biology, unavoidably generates or encounters a series of unfavorable circumstances during the culture process [20]. However, *C. glutamicum* robustly survives the various adverse stresses of the culture process using diverse defense strategies, including thiol-based regulator-control of a variety of antioxidants and MSH [11, 21–24]. However, surprising observations in previous reports showed that although MSH-deficient *C. glutamicum* mutants were highly sensitive to OHPs, MSH did not directly react with peroxides to protect the cells [11, 25, 26]. More recently, the *ohr* expression in a *C. glutamicum* strain lacking AhpC was found to be specifically induced by the OHPs and not regulated by the reactive electrophilic species (RES)-sensing zinc-associated anti sigma factors SigH, and *ohr* mutants were hypersensitive to the OHPs and were not sensitive to other ROS-generating agents [21]. In addition, in vitro biochemical studies showed that the thiol-dependent, Cys-based peroxidase Ohr could detoxify OHPs more effectively than H_2_O_2_ [21]. These recent discoveries raised questions about the existence of a specific Ohr-regulating and MSH-dependent response OHPs regulator. Although many H_2_O_2_-, quinone-, diamide-, or NaOCl-responding disulfide bond-based redox regulators, such as OxyR, RosR (regulator of oxidative stress response), QorR (quinone oxidoreductase regulator), and SigH (the stress-responsive extracytoplasmic function-sigma (ECF-σ) factor), have been investigated extensively in *C. glutamicum*, the study of transcriptional regulators specifically responding to organic oxidation and controlling *ohr* expression in *C. glutamicum* remains enigmatic, and very little is known about transcriptional regulators that use an *S*-mycothiolated regulation model [27–30].

In this study, we demonstrated that the regulator OhsR from *C. glutamicum* specifically sensed OHPs stress using a new thiol oxidation-based mechanism. The reduced OhsR did not bind to the Ohr promoters. The oxidative modification of Cys125 produced an intermediate Cys-SOH and reacted further with MSH to the mixed disulfides formation. *S*-mycothiolation caused the structural configuration of OhsR, which resulted in its functional activation and DNA-binding activity, eventually resulting in a strong activation of Ohr transcription. To the best of our knowledge, this is the first report demonstrating the ability of a MarR-type OhsR to specifically sense OHPs stress using an *S*-mycothiolated regulation model.

## Methods

### Strains and culture conditions

The strains and plasmids used in this study are listed in Additional file 1: Table S1. *E. coli* was cultivated at 37 °C in Luria–Bertani (LB) broth aerobically on a rotary shaker (220 rpm) or on LB agar plates. *C. glutamicum* was grown in LB at 30 °C as previously reported [31]. Five hundred millimolar sorbitol-containing brain–heart broth (BHIS) medium was used to generate and maintain the *C. glutamicum* mutants [31]. To construct the *ohsR* deletion mutants, the pK18*mobsacB*-Δ*ohsR* plasmids were transformed into the wild type (WT) *C. glutamicum* using electroporation to execute a single crossover. LB agar media containing 40 µg/mL nalidixic acid (Na) and 25 µg/mL kanamycin were used to select the transconjugants. A total of 40 µg/mL Na and 20% sucrose-containing LB agar plate was used to perform counter selection for the in-frame deletion [32]. Strains growing on Na and sucrose-containing plates were examined for kanamycin sensitivity (Km^S^) by parallel picking on 40 µg/mL Na-containing LB plates containing either 25 µg/mL kanamycin or 20% sucrose. Sucrose-resistant and kanamycin-sensitive strains were detected for *ohsR* deletion by PCR using the primer pair DOhsR-F1/DOhsR-R2 listed in Additional file 1: Table S2 and confirmed by DNA sequencing. For complementation, the pXMJ19 derivatives were used to transform the Δ*ohsR* mutants using electroporation. The expression of the corresponding gene in *C. glutamicum* was induced by the addition of 0.5 mM isopropyl β-D-1-thiogalactopyranoside (IPTG) to the media. To construct chromosomal fusion reporter strains, the suicide vector pK18*mobsacB*-*P*_*ohsR*_*::lacZ* was transferred into the relevant *C. glutamicum* strains using electroporation. The chromosomal pK18*mobsacB*-*P*_*ohsR*_*::lacZ* fusion strain was selected on LB agar plates with 25 µg/mL kanamycin and 40 µg/mL Na. Sensitivity assays for peroxides were performed as described [22]. Antibiotics were added at suitable concentrations: kanamycin, 50 µg/mL for *E. coli* and 25 µg/mL for *C. glutamicum*; nalidixic acid, 40 µg/mL for *C. glutamicum*; chloramphenicol, 20 µg/mL for *E. coli* and 10 µg/mL for *C. glutamicum*.

### Plasmid construction

To obtain an expression plasmid, the OOhsR-F/OOhsR-R primer pair listed in the Additional file 1: Table S2 and *C. glutamicum* genomic DNA was used to amplify the gene encoding OhsR (NCgl0024) by PCR. The amplified DNA fragments digested with *Bam*HI and *Sal*I were ligated to *Bam*HI- and *Sal*I-digested pET28a to obtain pET28a-*ohsR*.

The suicide plasmid pK18*mobsacB*-Δ*ohsR* was prepared using overlap PCR to construct the Δ*ohsR* deletion mutants [33]. Briefly, the primer pairs DOhsR-F1/DOhsR-R1 and DOhsR-F2/DOhsR-R2 (Additional file 1: Table S2) were used to obtain the 960 bp upstream fragments and 844 bp downstream fragments of *ohsR*, respectively. The primer pair DOhsR-F1/DOhsR-R2 (Additional file 1: Table S2) was used to fuse the upstream and downstream fragments together using overlap PCR [33]. The PCR fragments obtained were digested with *Eco*RI and *Bgl*II and inserted into the *Eco*RI- and *Bgl*II-digested pK18*mobsacB* to yield pK18*mobsacB*-Δ*ohsR*. The knock out plasmids pK18*mobsacB*-Δ*rosR* and pK18*mobsacB*-Δ*qorR* were constructed in a similar manner.

To prepare pXMJ19-*ohsR* and pXMJ19-His_6_-*ohsR*, the primer pair COhsR-F/COhsR-R (Additional file 1: Table S2) was used to amplify the PCR fragments of *ohsR* from the *C. glutamicum* genomic DNA. The PCR products of *ohsR* were digested with *Bam*HI and *Eco*RI and cloned into *Bam*HI- and *Eco*RI-digested pXMJ19 or pXMJ19-His_6_.

To obtain pK18*mobsacB*-*P*_*ohsR*_*::lacZ*, the *ohsR* promoter was fused into the *lacZY* reporter gene using overlap PCR. To amplify the 300 bp *ohsR* promoter fragments and the *lacZY* fragments, the primers POhsR-F1/POhsR-R1 and lacZY-F/lacZY-R were used in the first round of PCR, respectively. Second, the first round PCR fragments were used as a template with POhsR-F1/lacZY-R as primers in the second round of PCR. The resulting fragments were digested with *Sma*I and *Pst*I and cloned into *SmaI*- and *Pst*I-digested pK18*mobsacB* to obtain pK18*mobsacB*-*P*_*ohsR*_*::lacZ* [33]. DNA sequencing confirmed the reliability of all the constructs (Sangon Biotech, Shanghai, China).

To make the cysteine residue at position 125 of OhsR into a serine residue (OhsR:C125S), site-directed mutagenesis is performed [33]. Briefly, to obtain the mutant *ohsR:C125S* DNA segments, two rounds of PCR are used. In the first round of PCR, primer pairs DOhsR-F1/OhsR-C125S-R and OhsR-C125S-F/DOhsR-R2 were used to amplify segments 1 and 2, respectively. The second round of PCR was carried out using OhsR-F/OhsR-R as primers and fragment 1 and fragment 2 as templates to get the *ohsR:C125S* fragment. The *ohsR:C125S* DNA fragment was digested and cloned into digested pXMJ19 or pET28a plasmid, obtaining pXMJ19-*ohsR:C125S* or pET28a-*ohsR:C125S*. The *ohsR:C261S* fragments were gained using a similar procedure as described above and cloned into plasmid pXMJ19 or pET28a, obtaining pXMJ19-*ohsR:C261S* or pET28a-*ohsR:C261S*.

### Heterologous expression and purification of the recombinant proteins

To transform recombinant pET28a plasmids and purify the His_6_-tagged recombinant proteins, BL21(DE3) host strains were used. After the bacteria were cultivated at 37 °C in LB broth to an OD_600_ of 0.5, the cultures were transferred to 22 °C. Five hundred millimolar IPTG was added to induce the expression of the recombinant proteins. The resulting cultures continued to grow for an additional 10 h at 22 °C. After harvesting, the cells were disrupted by sonication, and the target proteins were purified using His•Bind Ni–NTA resin (Novagen, Madison, Wisconsin, USA) based on the manufacturer’s instructions target proteins eluted were dialyzed in Tris–HCl at 4 °C.

### Plate assay for peroxide sensitivity

Peroxide sensitivity was measured using an agar dilution method [11, 35]. *C. glutamicum* strains were cultivated at 30 °C in LB to an OD_600_ of 1.7 and subjected to serial tenfold dilutions with LB. A 5 μL portion from each dilution was plated onto LB plates with proper peroxide concentrations. The plates obtained were cultivated at 30 °C for 48 h before examination.

### Analysis of sulfenic acid formation

The formation of Cys-SOH in OhsR was measured using a 4-chloro-7-nitrobenzofurazan (NBD-Cl) labeling assay [36, 37]. After the proteins were pretreated with 50 mM dithiothreitol (DTT) for 30 min, the remaining DTT was removed by ultrafiltration. Proteins in the buffer (50 mM potassium phosphate with 1 mM EDTA at pH 7.0) were prepared anaerobically by repeated bubbling with argon gas and vacuum treatment for 20 min. A total of 25 mM NBD-Cl dissolved in dimethyl sulfoxide (DMSO) was bubbled with argon gas for 10 min to prepare an anaerobic solution. Under anaerobic conditions, the 50 μM proteins were treated with various concentrations of CHP, hydrogen peroxide (H_2_O_2_), or DTT (negative control). The resulting proteins were treated with 5 mM NBD-Cl at 25 °C for 30 min in the dark. The NBD-Cl remaining was eliminated by ultrafiltration, and the absorbance of the proteins was detected (200–600 nm) using a Beckman DU 7500 diode array spectrophotometer (Fullerton, California, USA).

### Quantitative analysis of sulfhydryl groups

Free sulfhydryl groups were detected using 5,5′-dithio-bis (2-nitrobenzoic acid) (DTNB) [38]. After OhsR WT and its variants (25 μM) were treated with 50 μM CHP and 50 mM DTT at room temperature for 30 min, respectively, a PD10 desalting column (GE Healthcare, Piscataway, New Jersey, USA) was used to remove residual DTT or CHP. The resulting proteins (10 μM) were cultured with 2 mM DTNB in 50 mM Tris–HCl buffer (pH8.0) and the absorbance at 412 nm was detected with a 2 mM DTNB solution as the reference. The quantities of reactive sulfhydryl groups were calculated with the molar absorption coefficient of TNB at 412 nm (*ε*_412_) of 13,600/M/cm [39].

### Protein thiol alkylation by polyethylene glycol (PEG)-maleimide and electrophoretic analysis

OhsR-SOH was prepared by immediately incubating the reduced OhsR (25 μM) with 25 μM CHP for 30 min. After 20 μM reduced Mrx1:C15S was added to the reaction mixtures following the oxidation step, 10 μL of trichloroacetic acid (TCA) (10% (w/v)) was added to the reduced and/or OhsR-SOH-containing reaction mixtures (100 μL) with or without MSH at the concentrations indicated and incubated on ice for 30 min to precipitate the proteins. The protein precipitates were washed with 100 μL ice-cold acetone, dried at 37 °C, resuspended in 15 μL of 3 mM PEG-maleimide (in 50 mM Tris, 10 mM EDTA, and 0.1% SDS, pH 7.5), and treated at 45 °C for 45 min. The resulting proteins were immediately separated on 15% sodium dodecyl sulfate polyacrylamide gel electrophoresis (SDS-PAGE) without β-Mercaptoethanol (β-ME) and stained with Coomassie Brilliant Blue (CBB).

### *S*-mycothiolation of OhsR in vivo

The *S*-mycothiolation states of OhsR were determined using the iodoacetamide (IAM)-biotin-tagged method as previously reported [24]. The IAM-alkylated protein extracts of the relevant strains were harvested before and 30 min after CHP stress and subjected to the His·Bind Ni–NTA resin purification. The resulting His_6_-OhsR was de-mycothionylated, and the free protein thiol was tagged with biotin-maleimide followed by separation on nonreducing 15% SDS-PAGE and blotted onto nitrocellulose membranes. The membranes were reacted with the HRP-conjugated streptavidin to detect the *S*-mycothiolation of OhsR and incubated with the anti-His antibody to indicate the same amount of His_6_-OhsR used for the de-mycothionylation analysis.

### MALDI-TOF MS–MS analysis

DTT-treated and CHP-treated OhsR were separated using nonreducing SDS-PAGE and stained with CBB. After staining, bands were excised, digested in-gel, and detected using matrix-assisted laser desorption ionization-time of flight tandem mass spectrometry (MALDI-TOF MS–MS) (Voyager-DE STR, Applied Biosystems, Madison, Wisconsin, USA).

### β-Galactosidase assay

β-Galactosidase activities were detected using *o*-nitrophenyl-β-d-Galactopyranoside galactopyranoside (ONPG) as the substrate [40].

### Electrophoretic mobility shift assay (EMSA)

The EMSA was conducted as previously described [23]. Briefly, a DNA promoter (*P*_*ohr*_; 200 bp) containing the predicted OhsR binding site was amplified from the *ohr* promoter region using the primers EOhr-F/EOhr-R (Additional file 1: Table S2). Increasing concentrations of purified OhsR (0–4 μg) were incubated with 20 ng *ohr* DNA promoter in EMSA buffer containing 20 mM Tris–HCl, pH 7.4, 4 mM MgCl_2_, 100 mM NaCl, and 10% glycerol. After the binding reaction mixture was preincubated at room temperature for 30 min, the mixture was separated on a 6% native polyacrylamide gel in 0.5 × TBE electrophoresis buffer, and the *ohr* promoter was detected using SYBR Green. BSA instead of OhsR in the binding assays was used as the negative control in the binding assays.

### Quantitative RT-PCR analysis

Isolation of total RNA and transcript levels analysis was performed as described previously [22].

### Peroxide scavenging by whole cells

Peroxide scavenging was detected as previously described with minor modifications [24]. Cultures grown in LB broth to an OD_600_ of 0.6 were divided and treated with either 0.75 mM CHP, 2.5 mM *t*-BHP, or 15 mM H_2_O_2_. At intervals, aliquots were immediately harvested, diluted when necessary, and assayed for peroxide contents using the FOX method [41]. The residual peroxide levels in the LB broth were measured to serve as a negative control.

### Western blot analysis

Western blot analysis was conducted as previously described [24]. The cytosolic RNA polymerase β was used as a control as previously reported [42]. The anti-OhsR and anti-Ohr rabbit polyclonal antibodies were produced and affinity-purified as previously described [24]. The density of the bands on the Western blots was quantified using Image Lab (Bio-Rad, California, USA).

## Results and discussion

### OhsR was required for the resistance to organic peroxides

To obtain homologs of the *B. subtilis* OhrR in the *C. glutamicum* proteome, a direct subsequent BLAST search was performed. No hits for the OhrR homologs in *C. glutamicum* were found, while one candidate that was annotated as a potential regulator due to the presence of the “winged helix” type of helix-turn-helix (HTH) motif encoded by the gene of *C. glutamicum* ATCC 13032 (*ncgl0024*) was found to be located adjacently downstream of the *ohr* gene. Although Ncgl0024 had a very low identity with *M. tuberculosis* MosR (*M. tuberculosis* oxidation-sensing regulator), *M. smegmatis* OhrR, *P. aeruginosa* OspR (oxidative stress response and pigment production regulator), *S. aureus* MgrA (multiple gene regulator A), and *B. subtilis* OhrR, it contained a conserved cysteine at position (Cys-125) presumed to act as an oxidation sensor and an additional cysteine (Cys-261) not conserved in OhrR/MgrA. In addition, Cys-125 was surrounded by many aromatic amino acids (Tyr-126, Phe-230, Phe-264, and Phe-265) and conserved in the oxidation-sensing transcriptional regulators [43] (Additional file 1: Figure S1). Therefore, we hypothesized that Ncgl0024 may control the expression of *ohr* and was an OhrR homolog. In this condition, we designated *ncgl0024* to be *ohsR* (organic hydroperoxides stress regulator).

**Fig. 1:**
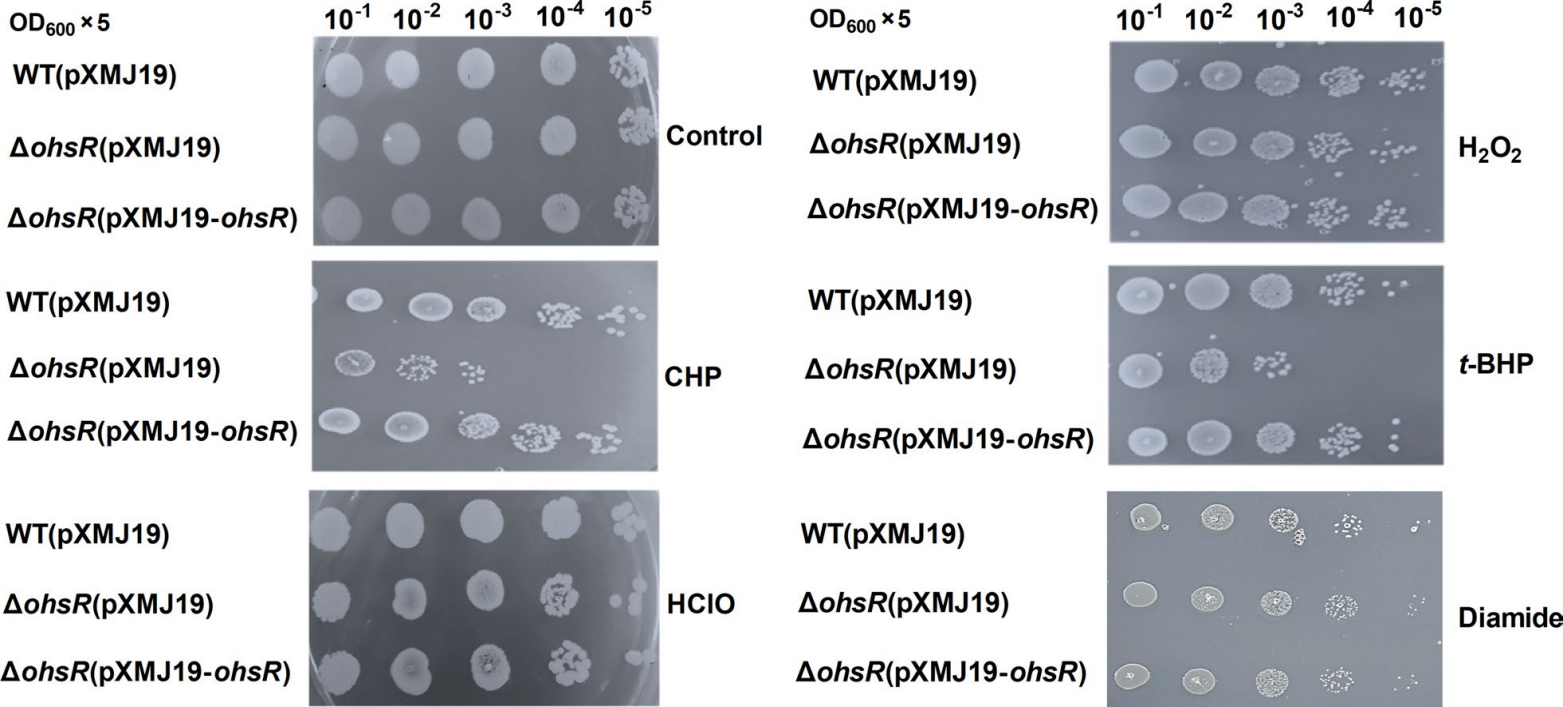
Plate sensitivity assay. WT(pXMJ19), Δ*ohsR*(pXMJ19) and Δ*ohsR*(pXMJ19-*ohsR*) strains were 5-times serially diluted from overnight growth cell suspension (an OD_600_ of 1.7) and spotted on LB agar plates supplemented without or with different peroxides at indicated concentrations and incubated at 30 °C for 48 h. The control plate had no peroxide. Similar results were obtained in three independent experiments, and data shown are from one representative experiment

OhrR was a redox sensor for OHPs, hypochlorous acid (HClO), H_2_O_2_, and superoxide anions [35, 44–46]. Therefore, the function of OhsR in the response to organic and inorganic oxidative stresses was studied using the plate sensitivity assay (Fig. 1). As shown in Fig. 1, the Δ*ohsR*(pXMJ19) strain (*ohsR* mutant with the empty plasmid pXMJ19) showed a marked increased in CHP and *t*-BHP sensitivity when compared to the WT(pXMJ19) strain (the wild type *C. glutamicum* strain with the empty plasmid pXMJ19). In addition, these relevant sensitive phenotypes could be restored with the wild-type *ohsR* gene inserted into a shuttle vector pXMJ19 expressed in the *ohsR* mutant (Δ*ohsR*(pXMJ19-*ohsR*)). There were no obvious differences between the Δ*ohsR* (pXMJ19) and WT(pXMJ19) strains when grown on plates containing inorganic peroxides, including H_2_O_2_, HClO, and diamide.

### Cys125 was first oxidized to sulfenic acid

The conserved cysteine of the *B. subtilis* OhrR was first oxidized to form a sulfenic acid (Cys-SOH) intermediate upon its reaction with CHP [36, 37]. Thus, we questioned whether oxidation could generate Cys-SOH in OhsR. To reveal the state of the Cys in OhsR under CHP treatment, the two variants, C125S and C261S, with each Cys mutated to Ser, respectively, were prepared, and the analytical methods of NBD-Cl and DTNB were used.

**Fig. 2:**
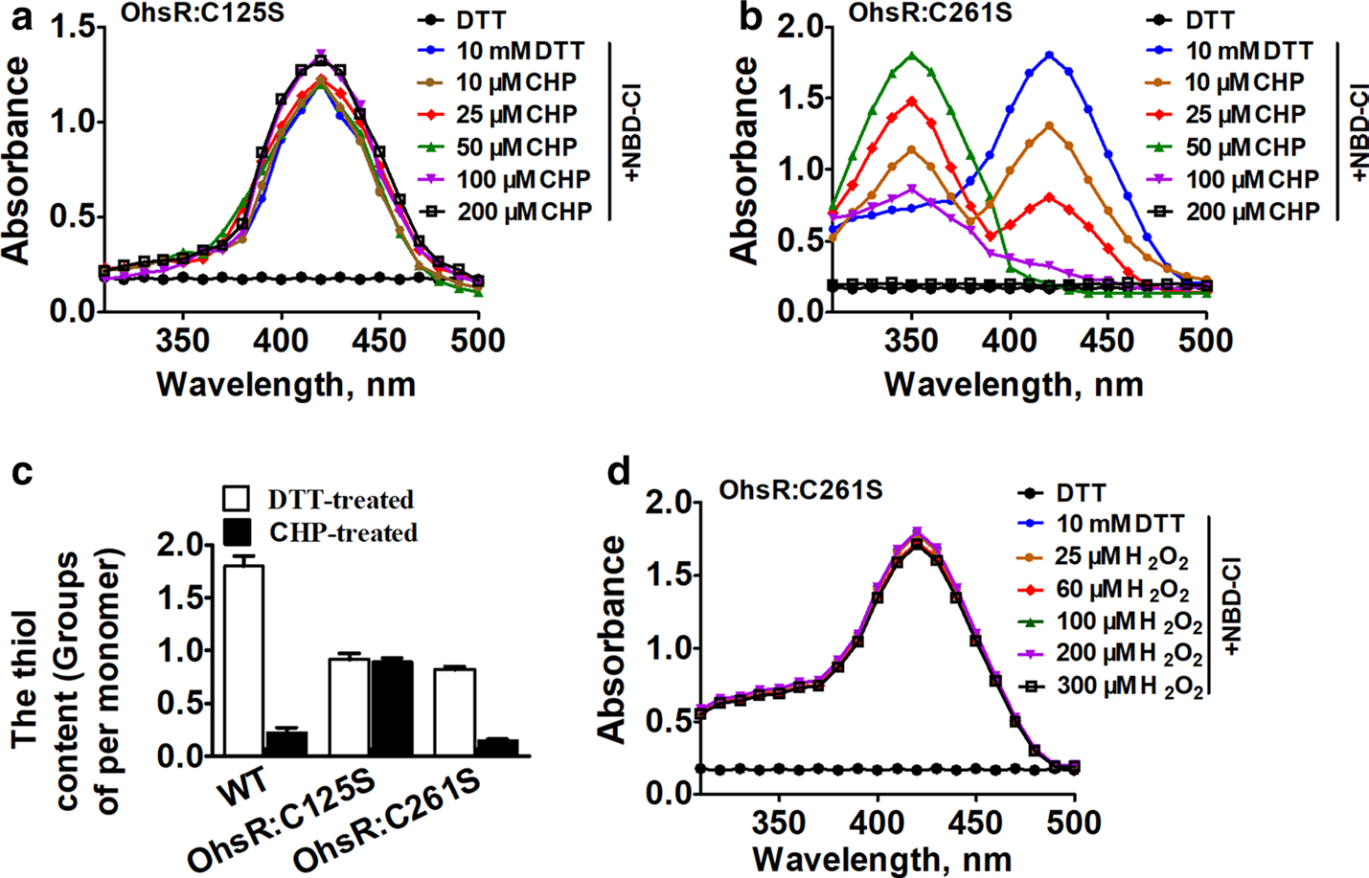
Cys125 was the peroxidative cysteine (C_P_). **a**, **b** Spectrophotometric analysis of NBD-labeled OhsR treated with or without different concentration of CHP. The proteins were analyzed spectrophotometrically at 200 to 600 nm. DTT indicates DTT-treated OhsR protein, which was as a negative control. **c** Quantification of free OhsR thiol levels in reduced and oxidized proteins. CHP- and DTT-treated proteins (10 μM) were mixed 2 mM with DTNB in 50 mM Tris–HCl buffer (pH 8.0), and the absorbance was monitored at 412 nm against a 2 mM DTNB solution as reference. These data are means of the values obtained from three independent assays. **d** Spectrophotometric analysis of NBD-labeled OhsR treated with or without different concentration of H_2_O_2_

NBD-Cl specifically reacts with sulfenic acids and thiol groups but not with sulfinic or sulfonic forms. The covalent attachment of NBD-Cl produced an exclusive absorption peak at approximately 420 nm when reacting with thiol groups, while a Soret band at approximately 347 nm occurred when reacting with sulfenic acids [36]. By reacting with NBD-Cl, the unchanged absorption spectra of the OhsR:C125S variants was observed upon exposure to different concentrations of CHP, only occurred at the 420 nm peak (Fig. 2a). OhsR:C125S variants, whether being treated with CHP or not, contained approximately 0.89 ± 0.2 thiol groups per monomer in the free thiol contents of the DTNB assay (Fig. 2c). These data indicated that there was no sulfenic acid in Cys261 and that Cys261 still existed in the thiol state in the CHP-treated OhsR:C125S variants.

Twenty-five micromolar OhsR:C261S upon treatment with less than 50 μM CHP showed a Soret band at 347 nm and 420 nm, indicating that the reaction of NBD-Cl with free thiol groups and sulfenic acids existed at the same time, and Cys125 was partially oxidized to a sulfenic acid form (Fig. 2b). The 50 μM CHP-treated and NBD-labeled OhsR had a specific absorbance maximum (λ_max_) of 347 nm, which clearly indicated that there were some stoichiometric amounts of detectable and trapping SOH at Cys125. The decrease or the disappearance of this signal upon treatment with more than 100 μM CHP indicated that Cys125 was probably overoxidized to the sulfinic or sulfonic acid forms. Consistently, the thiol content of the DTT-treated OhsR:C261S monomer was significantly higher than that of the CHP-treated OhsR:C261S monomer, and the difference of the 0.78 thiol content between the CHP-treated and the DTT-treated OhsR:C261S revealed that the CHP-treated OhsR:C261S contained no free thiol groups, indicating that Cys125 was susceptible to oxidation (Fig. 2c). However, after reacting with NBD-Cl, the absorption spectra of OhsR were unchanged before and after exposure to different concentrations of H_2_O_2_ (Fig. 2d), exhibiting only the 420 nm peak. These results suggest that the Cys125 residue was the peroxidative cysteine that can be oxidized to Cys-SOH under OHPs treatment.

### *S*-mycothiolated formation of OhsR in the presence of MSH upon organic peroxide oxidation

**Fig. 3:**
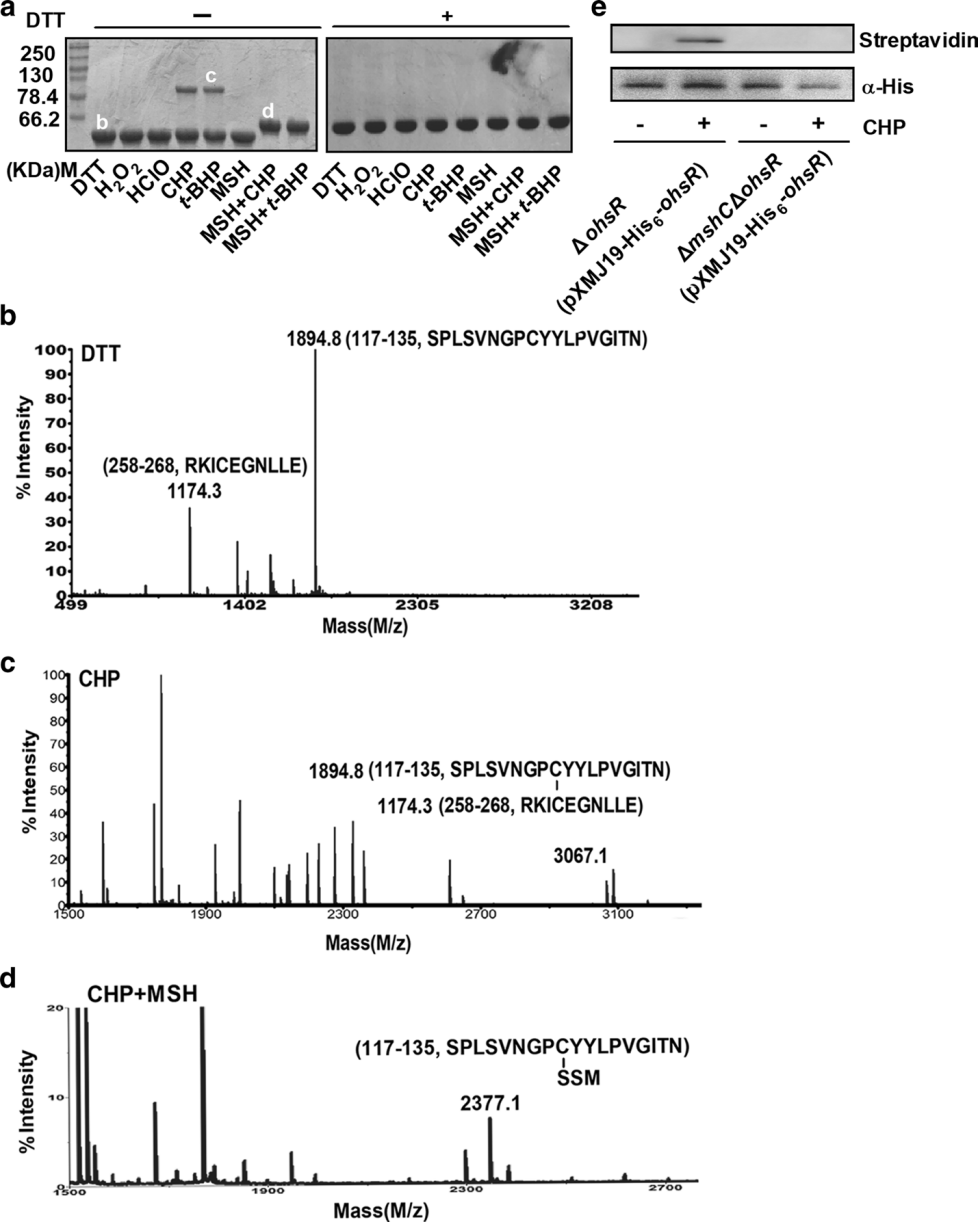
Redox response of OhsR detected by nonreducing SDS-PAGE. **a** 50 μM proteins treated with 50 mM DTT were further incubated with or without 150 μM H_2_O_2_, 75 μM HClO, 100 μM CHP, 200 μM *t*-BHP, 500 μM MSH, 150 μM CHP and 500 μM MSH, or 300 μM *t*-BHP and 500 μM MSH, and samples were then separated by 15% non-reducing SDS-PAGE (Left panel) and reducing SDS-PAGE (Right panel). **b**–**d** Analysis of *S*-mycothiolation and formed on OhsR by MALDI-TOF-TOF MS/MS. Bands (labeled (**b**–**d**)) excised from non-reducing gels in **a** were treated with trypsin and subjected to MS analysis. Only the relevant portion of each mass spectrum is shown. **e** *S*-mycothiolation of OhsR was monitored by biotin switch assay

Many OhrR regulators, such as *X. campestris* OhrR, *Sinorhizobium meliloti* OhrR, *M. tuberculosis* MosR, and *B. subtilis* OhrR, exist as intersubunit disulfide bond-containing homodimers, monomers containing an intramolecular disulfide bond, or *S*-bacillithiolation-containing monomers upon oxidation [18, 28, 35, 45, 46]. Thus, we hypothesized that it might share a similar oxidation-sensing mechanism, since OhsR was oxidized by one of the versatile thiol-disulfide switch manners. After OhsR was incubated with H_2_O_2_, HClO, CHP, *t*-BHP, or MSH, the resulting proteins were analyzed using nonreducing SDS-PAGE (Fig. 3a). Unexpectedly, on nonreducing SDS-PAGE, the purified OhsR not only was a monomer (Additional file 1: Figure S2) but H_2_O_2_- and HClO-treated OhsR also showed a single band with an apparent molecular weight (MW) of approximately 64 kDa. In addition, the electrophoretic migration states of the H_2_O_2_- and HClO-treated OhsR were the same as its DTT-treated state. However, OhsR exposed to CHP and *t*-BHP slightly showed the dimeric form as estimated by its behavior on 10% nonreducing SDS-PAGE. Even more surprising was that OhsR treated with CHP and MSH at the same time migrated more slowly than the form treated solely with MSH or DTT and lost the dimer morphology that existed in only the OHPs-treated OhsR (Fig. 3a). Importantly, in the presence of excessive DTT, the disappearance of dimerization and migration-delayed OhsR bands was observed, indicating that these bands can be reduced (Fig. 3a). Thus, we hypothesized that *S*-mycothiolation or intersubunit disulfide bonds were generated on Cys125 or between Cys125 and Cys261. This hypothesis was confirmed by peptide MALDI-TOF MS–MS after in-gel tryptic digestion of OhsR treated with CHP or CHP and MSH. A peak with a mass of 3067.1 Da occurred from the bands labeled with “c” produced through the Cys125-containing 117–135 peptide (calculated and observed mass 1894.8 Da) linking the Cys261-containing 258–268 peptide (calculated and observed mass 1174.3 Da) with a disulfide bond (Fig. 3b). A peptide mass of 2377.1 Da (Fig. 3d) that was 483.1 Da higher than the Cys125-containing 117–135 peptide of the reduced OhsR (calculated and observed mass 1894.8 Da) was identified (Fig. 3b), consistent with the result of adding MSH. However, the *S*-mycothiolated OhsR did not occur under only MSH treatment (Fig. 3a). The *S*-mycothiolated OhsR was also detected using a reversible biotinylation switch assay under the CHP treatment. The *C. glutamicum* strains Δ*ohsR*(pXMJ19-His_6_-*ohsR*) and Δ*mshC*Δ*ohsR*(pXMJ19-His_6_-*ohsR*) overexpressing His_6_-OhsR were treated with or without CHP stress. The purified His_6_-OhsR proteins from different cell extracts treated according to the biotin switch assay were pictured using Western blotting. As shown in Fig. 3d, His_6_-OhsR overexpressed in the Δ*ohsR*(pXMJ19-His_6_-*ohsR*) strain pretreated with CHP exhibited a strong band of *S*-mycothiolation. However, the *S*-mycothiolated signal did not appear in the CHP-untreated Δ*ohsR*(pXMJ19-His_6_-*ohsR*). Similarly, no *S*-mycothiolated signal was detected for His_6_-OhsR overexpressed in the Δ*mshC*Δ*ohsR*(pXMJ19-His_6_-*ohsR*) strain that does not produce MSH with or without CHP treatment (Fig. 3d). The result was consistent with that of Lee et al. who identified the *S*-bacillithiolation modification of *B. subtilis* MarR-type OhrR under CHP stress [17]. These results indicated that OhsR could react with MSH to undergo *S*-mycothiolation upon organic oxidative stresses.

### Reversible reaction of OhsR-SOH with MSH

**Fig. 4:**
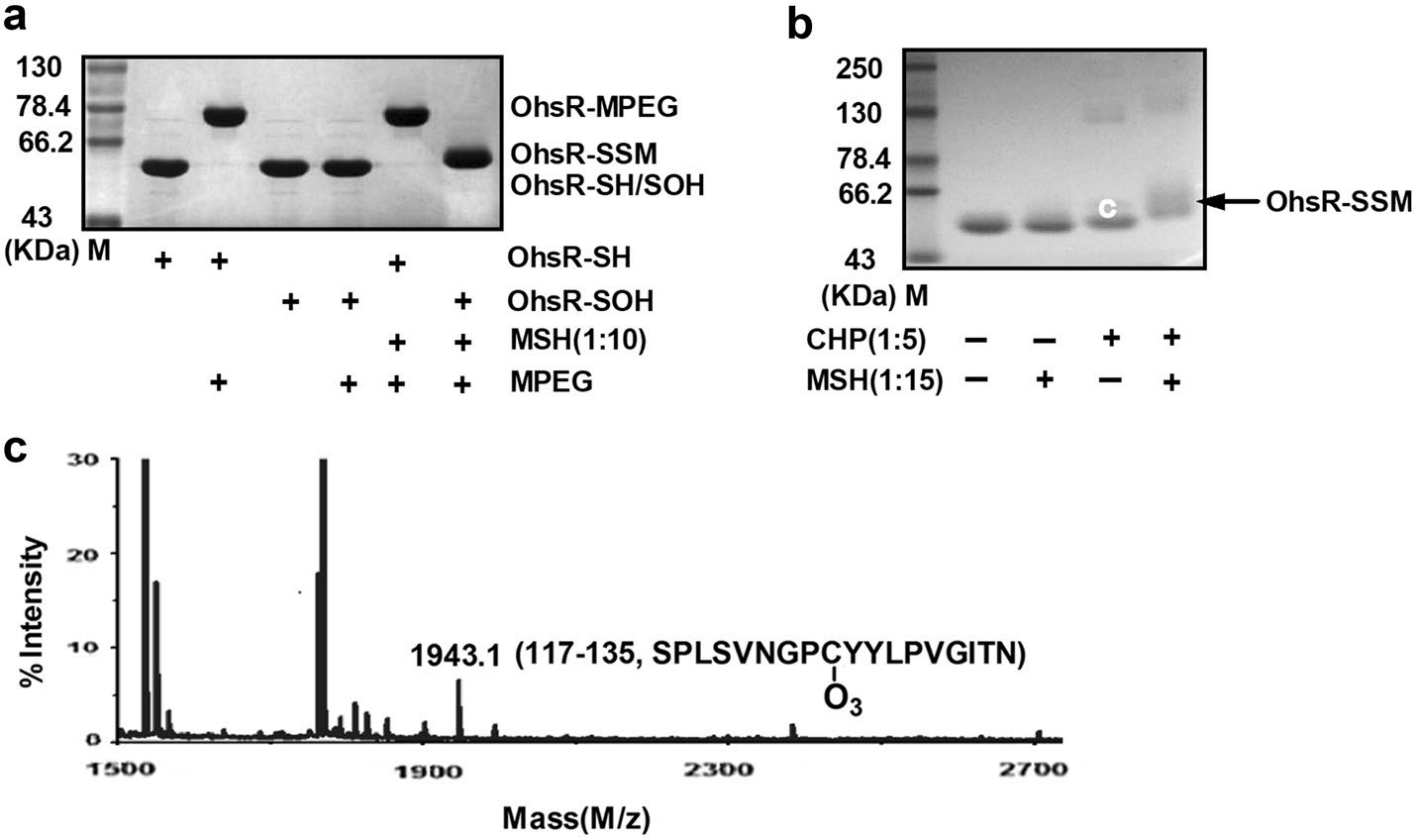
Reaction of OhsR-SOH with MSH but alkylation of OhsR with PEG-maleimide. **a** 20 μM reduced or OhsR-SOH was incubated with or without 200 μM MSH. Proteins were precipitated with TCA, treated with 5 mM PEG-maleimide, and evaluated on a CBB-stained 15% SDS-PAGE. **b** Proteins (50 μM) treated with 50 mM DTT were further incubated with or without 750 μM MSH, 250 μM CHP, or 250 μM CHP and 750 μM MSH, and samples were then separated by 15% nonreducing SDS-PAGE. **c** Analysis of sulfonic acid formed on OhsR by MALDI-TOF-TOF MS/MS. Bands (labeled (**c**)) excised from nonreducing gels in **b** were treated with trypsin and subjected to MS analysis. Only the relevant portion of each mass spectrum is shown

After the reduced ohsR was cultivated with excessive PEG-maleimide, the resulting proteins were analyzed using SDS-PAGE. As shown in Fig. 4a, a protein molecular weight shift of 5 kDa was detected, consistent with the addition of one molecule of PEG/protein and the existence of one thiol per reduced OhsR monomer. However, this observation of the molecular weight shift was not generated in OhsR-SOH that was formed from the initial treatment with CHP, which was consistent with the exclusive alkylation of the reduced Cys (Fig. 4a). Similarly, the thiol alkylation of OhsR-SOH was also not observed upon treatment with reduced MSH, indicating that only MSH could not reduce OhsR-SOH (Fig. 4a). However, the cultivation with MSH-protected the OhsR against oxidation-dependent dimerization and overoxidized sulfonic acid (Fig. 4b, c), because sulfonic acid formed on the catalytic Cys-125 was observed by peptide mass spectrometry after the tryptic digestion of CHP-treated OhsR with the identification of a mass of 1943.1 Da that was 48.3 Da higher than the Cys125-containing 117–135 peptides of the reduced OhsR (calculated and observed mass 1894.8 Da, Fig. 3b), consistent with the results from the addition of O_3_ (Fig. 4c). Considering that AhpE-SOH reacted with MSH by producing AhpE-SSM, we hypothesized that the protection was attributed to the OhsR-SSM formation.

### The expression of the *ohsR* genes was induced by organic peroxides

**Fig. 5:**
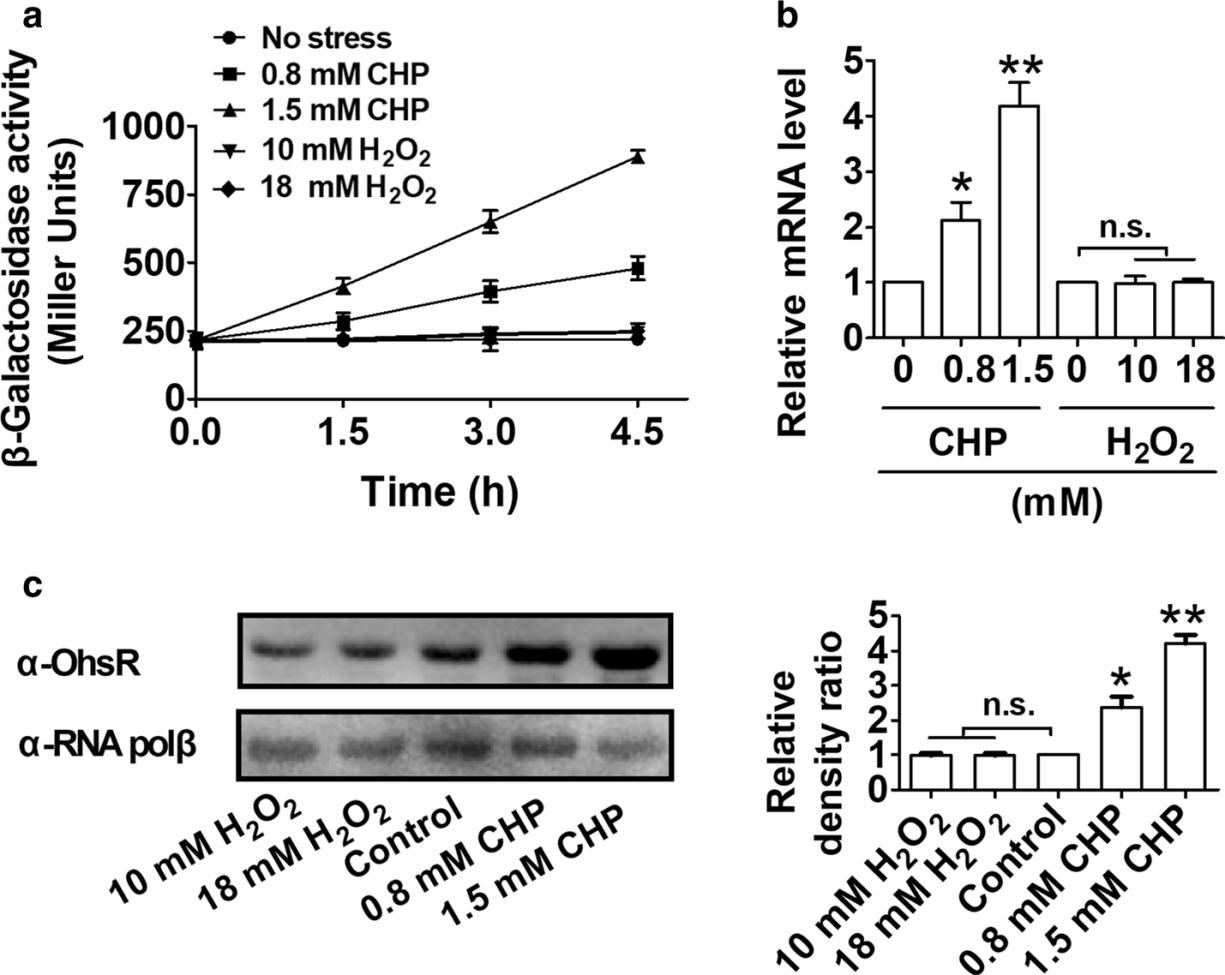
Induction of the expression of OhsR by CHP. Cells were grown in LB medium to an OD_600_ nm of 0.6. OhsR expression was analyzed after indicated CHP addition. **a** β-Galactosidase analyses of *ohsR* promoter activities by using the transcriptional *P*_*ohsR*_*::lacZ* chromosomal fusion reporter expressed in indicated strains under stress conditions. **b** Quantitative RT-PCR analyses of *ohsR* expression in indicated strains exposed to stress conditions for 4.5 h. Results were the average of four independent experiments; the standard deviation is indicated by bars. **P < 0.01; *P ≤ 0.05. *n.s*. not significant. **c** The protein levels of OhsR in wild type with or without peroxides treatment. Lysates from bacteria exposed to stress conditions for 4.5 h were resolved by SDS-PAGE, and OhsR was detected by immunoblotting using specific anti-OhsR antibody. For the pellet fraction, RNA polβ was used as a loading control. Similar results were obtained in three independent experiments, and data shown are from one representative experiment done in triplicate (Left panel). Relative quantified data for protein levels by Image Lab (Right panel). Quantified protein expression of western blots in **c**. Densities of proteins were all justified with α-RNA polβ. Relative density ratios of wild-type without stress were set at a value of 1.0. Data shown were the averages of three independent experiments, and error bars indicate the SDs from three independent experiments. **P < 0.01; *P < 0.05. *n.s.* not significant

To analyze the expression of the *ohsR* gene, the transcription levels of the chromosomal *P*_*ohsR*_::*lacZ* fusions in the WT strain were measured. The fusion expression of the strain growing to the exponential phase (OD_600_ of 0.6) in LB broth media was analyzed. The concentration of peroxides used did not influence the development of the exponentially growing WT cells (Additional file 1: Figure S3). *ohsR* expression was increased fourfold in the presence of 1.5 mM CHP (Fig. 5a). qRT-PCR analyses revealed that the *ohsR* expression was induced by CHP (Fig. 5b). In contrast, H_2_O_2_ did not affect the *ohsR* expression (Fig. 5a, b). Further analysis at the protein level indicated that a similar degree of regulation was observed for the protein production of OhsR, in which the CHP treatment increased its cellular level (Fig. 5c, Additional file 1: Figure S4).

### Positive regulation of *ohr* by OhsR

**Fig. 6:**
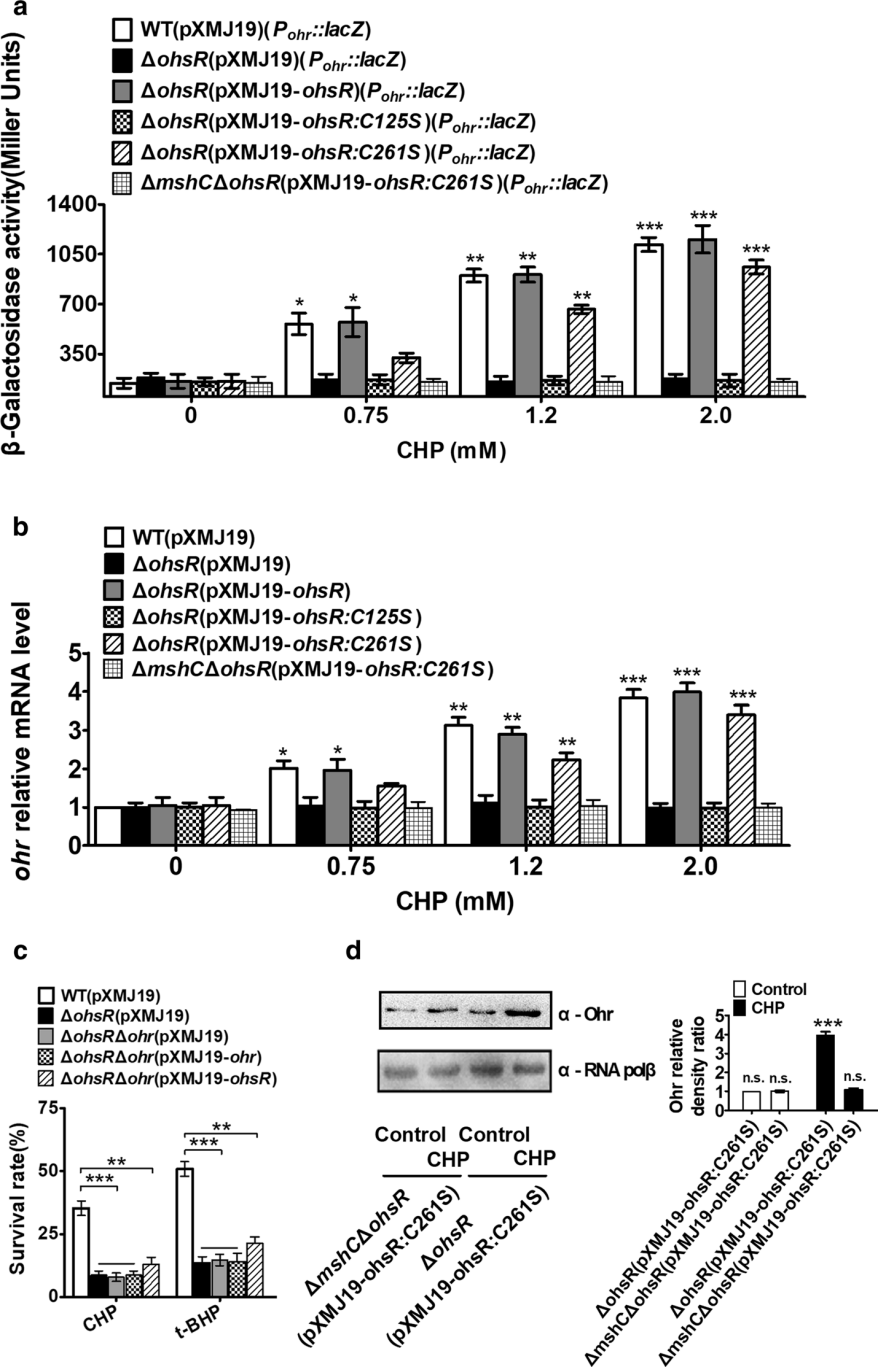
Positive regulation of *ohr* by OhsR. **a** β-Galactosidase analyses of *ohr* promoter activities by using the transcriptional *P*_*ohr*_*::lacZ* chromosomal fusion reporter expressed in indicated strains exposed to stress conditions for 30 min.***P < 0.001; **P < 0.01; *P ≤ 0.05. **b** Quantitative RT-PCR analyses of *ohr* expression in indicated strains exposed to stress conditions for 30 min. Results were the average of four independent experiments; the standard deviation was indicated by bars. ***P < 0.001; **P < 0.01; *P ≤ 0.05. **c** Survival rates for five relevant strains after challenged with 11 mM CHP and 20 mM *t*-BHP. Mean values with standard deviations (error bars) from at least three repeats were shown. ***P ≤ 0.001; **P < 0.01. **d** The protein levels of Ohr in Δ*ohsR*(pXMJ19-*ohsR:C261S*) and Δ*mshC*Δ*ohsR*(pXMJ19-*ohsR:C261S*), strains with or without CHP treatment. Lysates from stationary phase bacteria exposed to 1.5 mM CHP for 1 h were resolved by SDS-PAGE, and Ohr was detected by immunoblotting using specific anti-Ohr antibody. For the pellet fraction, RNA polβ was used as a loading control. Similar results were obtained in three independent experiments, and data shown are from one representative experiment done in triplicate (Left panel). Relative quantified data for protein levels by Image Lab (Right panel). Quantified protein expression of western blots in **d**. Densities of proteins were all justified with α-RNA polβ (beta chain of RNA polymerase). Relative density ratios of Ohr in the Δ*ohsR*(pXMJ19-*ohsR:C261S*) strain without stress were set at a value of 1.0. Data shown were the averages of three independent experiments, and error bars indicate the SDs from three independent experiments. **P < 0.01; *P < 0.05. *n.s.* not significant

Considering that the master H_2_O_2_-stress regulator in bacteria OxyR also regulated the AhpCF expression, we first examined whether *C. glutamicum* Ohr was regulated by the well-described peroxide-responding redox sensors OxyR, RosR, and QosR in *C. glutamicum* [27–29]. However, OxyR, RosR and QosR did not regulate Ohr (Additional file 1: Figure S5). It was previously noted that *C. glutamicum* Ohr was not regulated using the thiol-based redox sensor SigH [21]. Therefore, we hypothesized that OhsR might regulate *C. glutamicum* Ohr. The *lac*Z activity of the *P*_*ohr*_*::lacZ* chromosomal promoter fusion reporter in the relevant *C. glutamicum* strains and quantitative real-time PCR (qRT-PCR) profiling of *ohr* expression were measured in strains with or without different concentrations of CHP treatment. As shown in Fig. 6a, the *lacZ* activity under the driving of *ohr* promoter in the WT(pXMJ19) strain was enhanced in the presence of CHP compared to the untreated-CHP samples, and the expression levels of the *P*_*ohr*_::*lac*Z fusion displayed a dose-dependent increase in response to CHP. However, the promoter *lac*Z activity of *ohr* in the Δ*ohsR*(pXMJ19) strain under both CHP-treated and CHP-untreated conditions was very low, similar to that in the WT(pXMJ19) strain under CHP-untreated conditions. The low level of expression of *ohr* in the Δ*ohsR*(pXMJ19) strain was almost completely recovered in Δ*ohsR*(pXMJ19-*ohsR*) in the presence of CHP. These results indicated that the OhsR acted as a transcriptional activator of *ohr*. A similar regulatory pattern of *ohr* by the OhsR was also observed at the mRNA transcriptional level by using qRT-PCR analysis (Fig. 6b). Survival rate assays also showed that the Δ*ohr*Δ*ohsR*(pXMJ19) double mutants behaved identically to the *ohr* mutant in that they were less resistant than their parental strains when challenged with OHPs, and the Δ*ohr*Δ*ohsR* double mutants complemented by any of the *ohsR* and *ohr* wild type genes were still sensitive to OHPs, and their sensitivity levels were comparable with that of Δ*ohr*(pXMJ19) or Δ*ohsR*(pXMJ19) (Fig. 6c). Further analysis at the protein level showed that deletion of OhsR strongly decreased the cellular protein production of *ohr* under CHP treatment (Fig. 6d).

To further analyze the relevance of Ohr and OhsR in the response to OHPs *in C. glutamicum*, the residual concentration of peroxide in the culture medium was measured (Additional file 1: Figure S6). The Δ*ohr*(pXMJ19), Δ*ohsR*(pXMJ19), and Δ*ohr*Δ*ohsR*(pXMJ19) mutants showed a significantly reduced ability to detoxify OHPs (CHP and *t*-BHP), since after 4 h, the levels of the CHP and *t*-BHP decreased to some extent, which was slightly less than the culture not inoculated with bacteria but obviously higher than the culture with the wild type bacteria. In addition, the remaining amount of OHPs in the Δ*ohr*Δ*ohsR*(pXMJ19) mutants complemented by any of the *ohsR* and *ohr* wild type genes was still equivalent to that in Δ*ohr*(pXMJ19) or Δ*ohsR*(pXMJ19). These results indicated that the Ohr-OhsR system was a more efficient user of CHP and *t*-BHP compared to the other antioxidants that remove organic oxidants in *C. glutamicum*, such as MPx and Prx [22, 24], because the residual amounts of CHP and *t*-BHP were 16% for the wild type strain, but the Δ*ohr*(pXMJ19), Δ*ohsR*(pXMJ19), and Δ*ohr*Δ*ohsR*(pXMJ19) mutants only degraded approximately 26% of the hydroperoxides added (Additional file 1: Figure S6a, b). These profiles of OHPs consumption indicate that OhsR positively regulated Ohr. In contrast, Δ*ohr*(pXMJ19), Δ*ohsR*(pXMJ19) and Δ*ohr*Δ*ohsR*(pXMJ19) degraded H_2_O_2_ similarly to the wild type strain, indicating that Ohr and OhsR were not involved in the response to the inorganic oxidants (Additional file 1: Figure S6c). Taken together, these data indicate that Ohr/OhsR was the primary pathway for *C. glutamicum* to specifically degrade OHPs.

### OhsR was bound to DNA by a *S*-mycothiolation mechanism

**Fig. 7:**
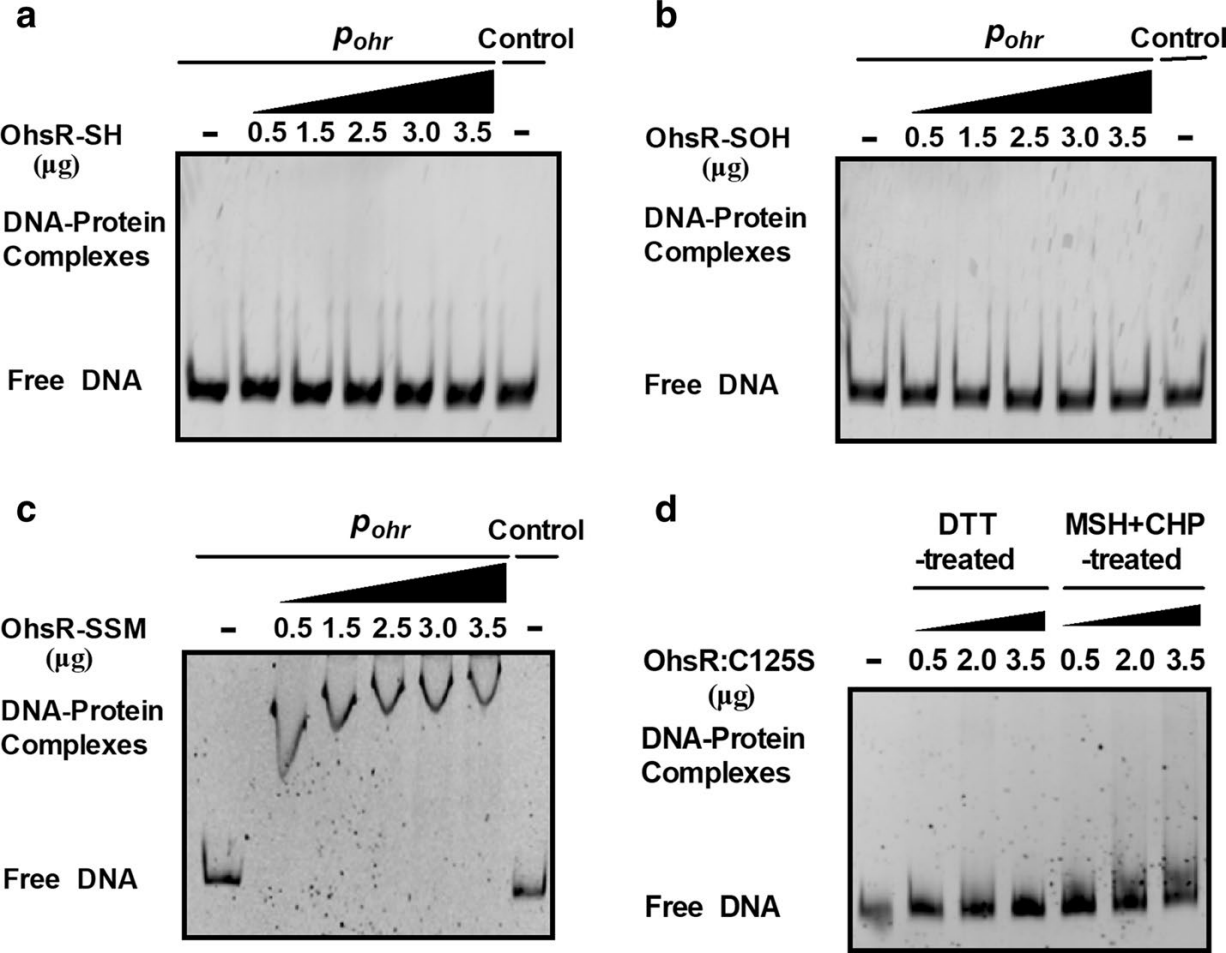
*S*-mycothiolated OhsR bound to the *ohr* promoter DNA. **a**–**c** Binding of reduced OhsR, OhsR-SOH, and OhsR-SSM to the *ohr* promoter examined by EMSA. Interaction of the increasing amounts of OhsR with the *ohr* promoter was detected using gel shift experiments. BSA instead of OhsR (Last lane) in the binding assays was used as negative controls to determine the binding specificity of OhsR. **d** EMSA experiment of interactions between DTT-treated OhsR:C125S or CHP- and MSH-treated OhsR:C125S and the *ohr* promoter was determined as described above. Similar results were obtained in three independent experiments, and data shown are from one representative experiment done in triplicate

Based on the number of Cys residues, the OhrR family can be divided into two subfamilies: the one-Cys-type OhrR and the two-Cys-type OhrR. Since the 1-Cys-type or 2-Cys-type OhrR regulators, including *B. subtilis* OhrR, *S. aureus* MgrA (multiple gene regulator A), *S. aureus* SarZ (a redox active global regulator), *X. campestris* OhrR, and *S. meliloti* OhrR, fulfilled the regulatory role via *S*-bacillithiolation, Cys-SOH, or disulfide bond mechanism, we subsequently tested the mechanism used by OhsR to regulate gene expression [17, 47–49]. Because OhsR existed in the form of *S*-mycothiolation in the presence of MSH under CHP treatment in vitro and in vivo (Fig. 3a, lane 7, e). Therefore, OhsR-SOH and OhsR-SSMwere prepared, which were confirmed using NDB-Cl, DTNB, AMS, and MALDI-TOF MS–MS assays (Figs. 3, 4). EMSA was performed by the interaction of the reduced (DTT-treated) OhsR, OhsR-SOH, or OhsR-SSM, with the *ohr* promoter region. As shown in Fig. 7a, the reduced OhsR did not form a complex with *P*_*ohr*_, a 200 bp PCR fragment amplified from the *ohr* promoter. OhsR was oxidized to sulfenic acid at Cys125 but still retained non-DNA binding activity, similar to the results of a 200 bp control DNA amplified from the *ohr* coding region showing undetectable OhsR binding (Fig. 7b). However, OhsR-SSM had the ability to bind *P*_*ohr*_, because the incubation of OhsR-SSM with *P*_*ohr*_ caused retarded mobility of the DNA (Fig. 7c, d). To further determine whether *S*-mycothiolated OhsR was required for the *ohr* expression in vivo, we treated cells of the Δ*ohsR*(pXMJ19-*ohsR:C261S*) andΔ*mshC*Δ*ohsR*(pXMJ19-*ohsR:C261S*) strains with 1.5 mM CHP, respectively, and probed the cellular protein production of Ohr by immunoblotting after the nonreducing SDS-PAGE separation (Fig. 6d). As expected, the Ohr in the Δ*ohsR*(pXMJ19-*ohsR:C261S*) strains exposed to CHP showed a significant increase in their cellular protein levels compared to the untreated samples, while the cellular protein production of Ohr under CHP treatment was not enhanced in the Δ*mshC*Δ*ohsR*(pXMJ19-*ohsR:C261S*) strains compared to the untreated samples. Consistently, in the case of the CHP concentration applied that was unable to reduce the survival rate of the relevant strains (Additional file 1: Figure S7), the promoter *lac*Z activity of *ohr* in the Δ*ohsR*(pXMJ19-*ohsR:C261S*) strains significantly increased in the CHP treatment, while there was no enhancement of the *ohr* lacZ activity in the Δ*mshC*Δ*ohsR*(pXMJ19-*ohsR:C261S*) strains exposed to CHP compared to the untreated samples (Fig. 6a, b). This important result indicated that OhsR-SSM obtained its affinity for DNA, which might lead to the activation of Ohr.

### Cys125 was critical for the DNA binding of OhsR under CHP conditions

The studies described above indicated that the OhsR-SSM bound to the target promoters and activated transcription (Fig. 7). In the presence of OHPs, OhsR was activated and bound to the promoter. It was of interest to know whether Cys125 of OhsR played an extremely important role in the OHPs-sensing and activation mechanisms. Thus, the ability of the OhsR:C125S variant to activate *ohr* expression in response to CHP was evaluated in the Δ*ohsR* (pXMJ19) strain background using the promoter *lac*Z activity and qRT-PCR analysis. Analysis of the *lacZ* activity revealed that Δ*ohsR*(pXMJ19-*ohsR:C125S*) did not activate the *ohr* expression under CHP-treated conditions to equal degrees, similar to the Δ*ohsR*(pXMJ19) strain, indicating that the cysteine had a role in the binding of OhsR to the *ohr* promoter under CHP-treated conditions (Fig. 6a**)**. In contrast, the Δ*ohsR*(pXMJ19-*ohsR:C261S*) strains had a clearly positive effect on the ability of CHP to activate the expression of *ohr*, consistent with the observation that the promoter *lac*Z activity of *ohr* in the WT (pXMJ19) strain was significantly increased with the increase in the CHP concentration (Fig. 6a). A similar regulatory pattern of *ohr* expression in the Δ*ohsR*(pXMJ19-*ohsR:C261S*) strain exposed to CHP was also obtained using the qRT-PCR experiments (Fig. 6b). The results implied that the Cys125 residue had a critical function in the OhsR sensing process.

To further probe whether the Cys125 residue was responsible for the observed combination of OhsR under oxidation, we pretreated OhsR:C125S with 0.1 mM CHP and 500 µM MSH for 90 min to perform the EMSA experiment. As shown in Fig. 7d, CHP- and MSH-treated OhsR:C125S did not produce any obvious retarded mobility, indicating that OhsR:C125S completely failed to provide the CHP-mediated combination of OhsR.

## Conclusions

Thiol-based redox-responsive regulators are recognized as a vital and efficient device to combat ROS-inducing diverse stress conditions in bacteria. Among these, the MarR family is one of the most common of the regulator families. In this study, we verified that the MarR-type regulatory OhsR exclusively sensed organic peroxide stress and responded to it by activating a specific *ohr* gene under its control with a *S*-mycothiolation model. Mutational analysis of the two cysteines (Cys125 and Cys261) in OhsR showed that Cys125 was critical for the activation of the DNA binding. This investigation provided a new perspective on stress responses in this and related human pathogens.

## Additional file

**Additional file 1: Table S1.** Bacterial strains and plasmids used in this study. **Table S2.** Primers used in this study. **Figure S1** Multiple sequence alignment of OhsR with the OhrR in other organisms. **Figure S2** Non-reducing SDS-PAGE analysis of proteins expressed in *E. coli* containing pET28a-*ohsR* plasmid. **Figure S3** Growth curves of *C. glutamicum* in response to sub-lethal concentrations of toxins. **Figure S4** The OhsR and Ohr was examined in *C. glutamicum*. **Figure S5** Regulation of *ohr* by OxyR, QorR, and RosR. **Figure S6** Role of OhsR in scavenging peroxides. **Figure S7** Survival rates of the relevant *C. glutamicum* in response to the different concentrations of CHP.

## Abbreviations

ROS: reactive oxygen species
OHPs: organic peroxides
Ohr: organic hydroperoxide reductase
CHP: cumene hydroperoxide
*t*-BHP: *t*-butyl hydroperoxide
H_2_O_2_: hydrogen peroxide
MSH: mycothiol
GSH: tripeptide glutathione
BSH: bacillithiol
MD: menadione
HTH: helix-turn-helix
NBD-Cl: 4-chloro-7-nitrobenzofurazan
DTT: dithiothreitol
DMSO: dimethyl sulfoxide
DTNB: 5,5′-dithio-bis (2-nitrobenzoic acid)
TCA: trichloroacetic acid
CBB: Coomassie Brilliant Blue
AhpCF: alkyl hydroperoxide reductase subunit CF
