# Supplementary material for: OhsR acts as an organic peroxide-sensing transcriptional activator using an S-mycothiolation mechanism in Corynebacterium glutamicum
Source: Microb Cell Fact. 2018 Dec 26;17:200. doi: 10.1186/s12934-018-1048-y (PMC6306002; doi:10.1186/s12934-018-1048-y)
Supplement: Supplementary file 1 — Additional file 1: Table S1. Bacterial strains and plasmids used in this study. Table S2. Primers used in this study. Figure S1 Multiple sequence alignment of OhsR with the OhrR in other organisms. Figure S2 Non-reducing SDS-PAGE analysis of proteins expressed in E. coli containing pET28a-ohsR plasmid. Figure S3 Growth curves of C. glutamicum in response to sub-lethal concentrations of toxins. Figure S4 The OhsR and Ohr was examined in C. glutamicum. Figure S5 Regulation of ohr by OxyR, QorR, and RosR. Figure S6 Role of OhsR in scavenging peroxides. Figure S7 Survival rates of the relevant C. glutamicum in response to the different concentrations of CHP. [file 12934_2018_1048_MOESM1_ESM.docx]

**Additional Data**

**OhsR acts as an organic peroxide-sensing transcriptional activator using an *S*-mycothiolation mechanism in *Corynebacterium glutamicum***

Meiru Si^1#*^, Tao Su^1#^, Can Chen^2#*^, Jinfeng Liu^1^, Zhijin Gong^1^, Chengchuan Che^1^, GuiZhi Li^1^, Ge Yang^1^

^1^ College of Life Sciences, Qufu Normal University, Qufu, Shandong 273165, China;

^2^ College of Life Science and Agronomy, Zhoukou Normal University, Zhoukou，Henan 466001, China

Running title: OhsR is organic peroxide-specific redox sensor

**^#^** These authors contributed equally to this work.

**^*^** Corresponding authors:

Meiru Si, Can Chen

E-mail: [smr1016@126.com](mailto:smr1016@126.com); [chenc02@126.com](mailto:chenc02@126.com)

Tel: 86-15666758564; 86-18736207816

**Table S1. Bacterial strains and plasmids used in this study.**

| **Strains or plasmids** | **Relevant genotype description** | **References** | |
| --- | --- | --- | --- |
| **Strains** | | | |
| ***Corynebacterium glutamicum*** | | | |
| RES167 | Restriction-deficient mutant of ATCC13032, Δ(*cglIM-cglIR-cglIIR*) | | [1] |
| Δ*ohsR* | *ohsR* deleted in RES167 | | This study |
| WT(pXMJ19) | wild type RES167 containing pXMJ19 vector | | [2] |
| Δ*ohsR*(pXMJ19) | Δ*ohsR* containing pXMJ19 vector | | This study |
| Δ*ohsR*(pXMJ19-*ohsR*) | Complementation of *ohsR* in Δ*ohsR* | | This study |
| Δ*ohsR*(pXMJ19-*ohsR:C125S*) | Complementation of *ohsR:C125S* in Δ*ohsR* | | This study |
| Δ*ohsR*(pXMJ19-*ohsR:C261S*) | Complementation of *ohsR:C261S* in Δ*ohsR* | | This study |
| Δ*ohsR* (pXMJ19-His_6_-*ohsR*) | Δ*ohsR* containing pXMJ19-His_6_-*ohsR* | | This study |
| Δ*mshC*Δ*ohsR* (pXMJ19-His_6_-*ohsR*) | Δ*mshC*Δ*ohsR* containing pXMJ19-His_6_-*ohsR* | | This study |
| Δ*mshC*Δ*ohsR*(pXMJ19-*ohsR:C261S*) | Complementation of *ohsR:C261S* in Δ*mshC*Δ*ohsR* | | This study |
| Δ*ohr* | *ohr* deleted in RES167 | | [3] |
| Δ*ohr*Δ*ohsR* | *ohr* and *ohsR* deleted in RES167 | | This study |
| Δ*oxyR* | *oxyR* deleted in RES167 | | [4] |
| Δ*sigH* | *sigH* deleted in RES167 | | [3] |
| Δ*rosR* | *rosR* deleted in RES167 | | This study |
| Δ*qorR* | *qorR* deleted in RES167 | | This study |
| ***E. coli*** | | | |
| BL21(DE3) | Host for expression vector pET28a | | Novagen |
| JM109 | *recA1 supE44 endA1 hsdR17 gyrA96 relA1 thi* Δ(*lac-proAB*)F′(*traD36 proABlacI*^q^ *lacΔZM15*) | | Stratagene |
| **Plasmids** | | | |
| pK18*mobsacB* | Suicide plasmid carrying *sacB* for selecting double crossover in *C. glutamicum*, Km^r^ | | [5] |
| pK18*mobsacB-*Δ*ohsR* | Construct used for in-frame deletion of *ohsR* | | This study |
| pK18*mobsacB-P_ohsR_::lacZ* | *P_ohsR_::lacZ* fusion in pK18*mobsacB* | | This study |
| pK18*mobsacB-P_ohr_::lacZ* | *P_ohr_::lacZ* fusion in pK18*mobsacB* | | [3] |
| pXMJ19 | Shuttle vector (*Ptac lacI^q^ pBL1 oriV_C. glutamicum_* pK18 *oriV_E. coli_*) | | [6] |
| pXMJ19-*ohsR* | *ohsR* cloned into pXMJ19 for complementation | | This study |
| pXMJ19-*ohsR:C125S* | *ohsR:C125S* cloned into pXMJ19 for complementation | | This study |
| pXMJ19-*ohsR:C261S* | *ohsR:C261S* cloned into pXMJ19 for complementation | | This study |
| pXMJ19-His_6_-*ohsR* | *ohsR* cloned into pXMJ19-His_6_ for complementation | | This study |
| pET28a | Expression vector with N-terminal hexahistidine affinity tag | | Novagen |
| pET28a*-trx* | *trx* in pET28a | | [7] |
| pET28a*-trxR* | *trxR* in pET28a | | [7] |
| pET28a*-ohsR* | *ohsR* in pET28a | | This study |
| pET28a*-ohsR:C125S* | *ohsR:C125S* in pET28a | | This study |
| pET28a-*ohsR:C261S* | *ohsR:C261S* in pET28a | | This study |

**Additional References**

1. Tauch A, Kirchner O, Löffler B, Götker S, Pühler A, Kalinowski J. Efficient electrotransformation of corynebacterium diphtheriae with a mini-replicon derived from the *Corynebacterium glutamicum* plasmid pGA1. [Curr Microbiol](http://www.ncbi.nlm.nih.gov/pubmed/?term=Efficient+electrotransformation+of+corynebacterium+diphtheriae+with+a+mini-replicon+derived+from+the+Corynebacterium+glutamicum+plasmid+pGA1.). 2002; 45: 362-367.

2. Schäfer A, Tauch A, Jäger W, Kalinowski J, Thierbach J, Pühler A. Small mobilizable multi-purpose cloning vectors derived from the *Escherichia coli* plasmids pK18 and pK19: selection of defined deletions in the chromosome of *Corynebacterium glutamicum*. Gene. 1994;145: 69-73.

3. Si M, Wang J, Xiao X, Guan J, Zhang Y, Ding W, Chaudhry MT, Wang Y, Shen X. Ohr Protects *Corynebacterium glutamicum* against organic hydroperoxide induced oxidative Stress. PLoS One. 2015;10(6): e0131634.

4. Si M, Wang T, Pan J, Lin J, Chen C, Wei Y, Lu Z, Wei G, Shen X. Graded response of the multifunctional 2-Cysteine peroxiredoxin, CgPrx, to increasing levels of hydrogen peroxide in *Corynebacterium glutamicum*. Antioxidants & Redox Signaling. 2017;26(1):1-14.

5. Jakoby M, Ngouoto-Nkili CE, Burkovski A. Construction and application of new *Corynebacterium glutamicum* vectors. Biotechnol Tech.1999;13: 437-441.

6. Karimova G, Pidoux J, Ullmann A, Ladant D. A bacterial two-hybrid system based on a reconstituted signal transduction pathway. [Proc Natl Acad Sci U S A](http://www.ncbi.nlm.nih.gov/pubmed/9576956).1998;95;5752-5756.

7. Su T, Si M, Zhao Y, Liu Y, Yao S, Che C, Chen C. A thioredoxin-dependent peroxiredoxin Q from *Corynebacterium glutamicum* plays an important role in defense against oxidative stress. PLoS One.2018;13(2*)*: e0192674.

Table S2. Primers used in this study.

| **Primers** |  | **5’-3’ sequence** |  |
| --- | --- | --- | --- |
| COhsR-F |  | CGCGGATCGTGGAACCATTCGAATTAGAGAAAG(*Bam*HI) | For cloning *ohsR* and its variants into pXMJ19 and pXMJ19-His_6_ |
| COhsR-R |  | CCGGAATTCTCAAGCCTTCAAGTACGCTCGGTTG(*Eco*RI) |  |
| OOhsR-F |  | CGCGGATCGTGGAACCATTCGAATTAGAGAAAG(*Bam*HI) | For cloning *ohsR* and its variants into pET28a |
| OOhsR-R |  | ACGCGTCGACTCAAGCCTTCAAGTACGCTCGGTTG(*Sal*I) |  |
| DOhsR-F1 |  | CCGGAATTCGCAACGGCGCCGTCGTATTCAACG (*Eco*RI) | To generate pK18*mobsacB-*Δ*ohsR* and *ohsR* mutant DNA fragments |
| DOhsR-R1 |  | GATTTCACTTCTACGGTTTCATCG |  |
| DOhsR-F2 |  | CGATGAAACCGTAGAAGTGAAATCGGTTGCAACTGCTCCACCATCAAG |  |
| DOhsR*-*R2 |  | GGAAGATCTGGGCCGGGATTTTTGTTGTTTAG (*Bgl*II) |  |
| POhsR-F |  | TCCCCCGGGGAACCCCCAACGAGCTCACCGGAG(*Sma*I) | To generate pK18*mobsacB-P_OhsR_::lacZ* |
| POhsR-R |  | ACTAGTTTCGAATGGTTCCACGGTGGCGAGTG(*Spe*I) |  |
| lacZY-F |  | CACTCGCCACCGTGGAACCATTCGAAACTAGTATGACCATGATTACGGATTC(*Spe*I) |  |
| lacZY-R |  | AAAACTGCAGTTAAGCGACTTCATTCACCTG(*Pst*I) |  |
| OhsR-C125S-F |  | GTGAACGGGCCC*A*GTTATTATCTTC | To obtain pET28a-*ohsR:C125S* and pXMJ19-*ohsR:C125S* |
| OhsR-C125S-R |  | GAAGATAATAAC*T*GGGCCCGTTCAC |  |
| OhsR-C261S-F |  | GACCGAAAAATC*A*GCGAGGGAAATC | To generate pET28a-*ohsR:C261S* and pXMJ19-*ohsR:C261S* |
| OhsR-C261S-R |  | GATTTCCCTCGC*T*GATTTTTCGGTC |  |
| QOhsR-F |  | CTTGCTCACCCGCCTTGACG | RT-PCR |
| QOhsR-R |  | AGTTTAGCGGGGCATGCTTG |  |
| QOhr-F |  | CCGCCAAAGGCTTTGGGCGG | RT-PCR |
| QOhr-R |  | GCTAACTCGGGCACCAACCG |  |
| EOhr-F |  | CACCTTGTTGGTGGGCCGGG | To produce the 200 bp EMSA Ohr promoter DNA |
| EOhr-R |  | GAGAGAACACAAAGAATACG |  |
| Control-F |  | GGTGCGCTCGAATTTGAAATG | To produce the 200 bp EMSA control DNA of Ohr |
| Control-R |  | CCACCAGCGCCGTTTGGCCC |  |

Underlined sites indicate restriction enzyme cutting sites added for cloning. Letters in italic denote the mutation sites in overlap PCR for site-directed mutation.


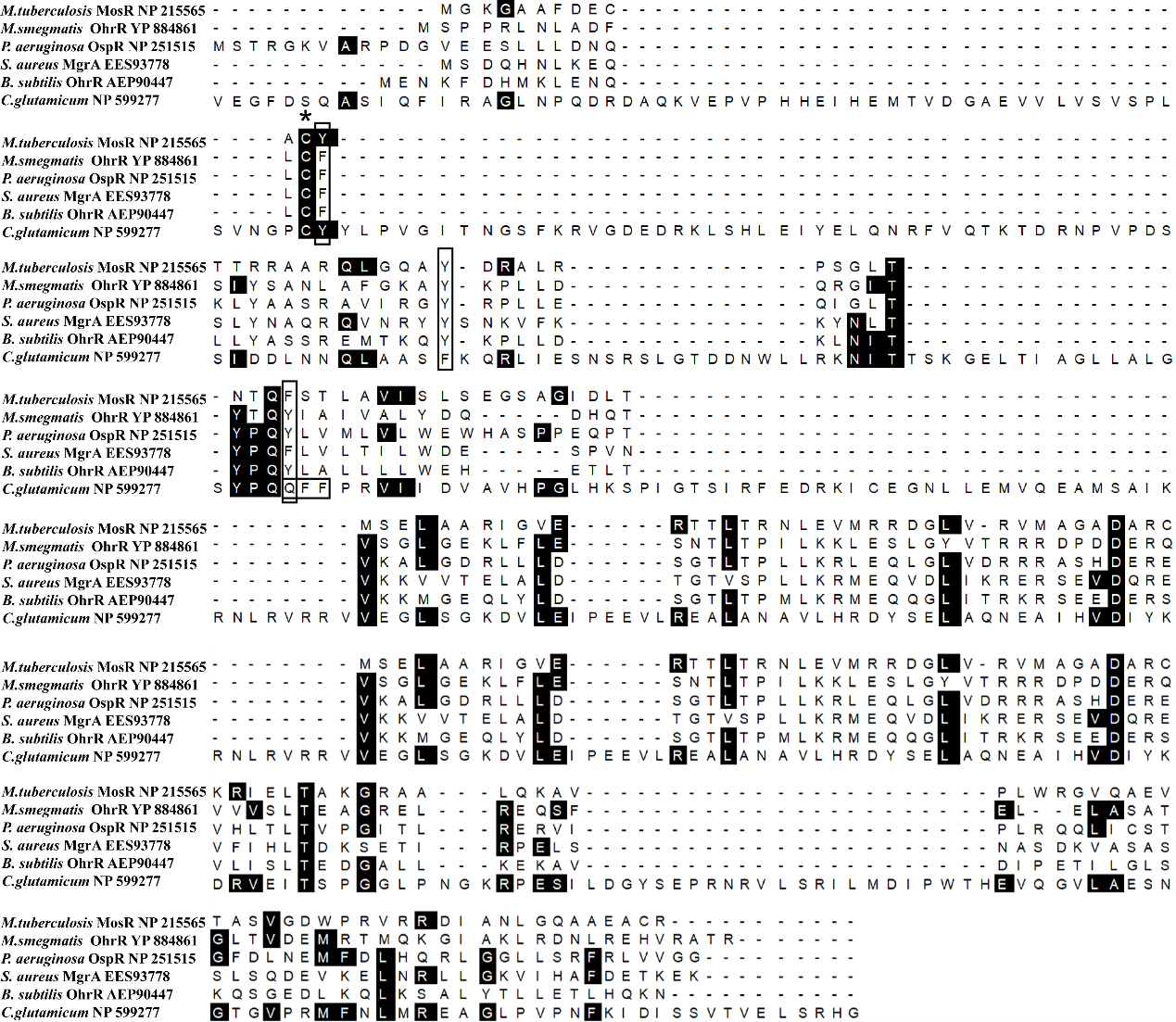


**Figure S1 Multiple sequence alignment of OhsR with the OhrR in other organisms.** Conserved Cys residue was highlighted in a black star. Aromatic amino acids surrounding conserved Cys residue were pointed out by black box. Residues that were identical in all of the 6 sequences were depicted on the black background. Reference sequences were retrieved from the NCBI Database, including *M. tuberculosis* MosR (NP 215565); *M. smegmatis* OhrR (YP 884861); *P. aeruginosa* OspR (NP 251515); *S. aureus* MgrA (EES93778); *B. subtilis* OhrR (AEP90447), *C. glutamicum* ATCC (NP 599277).


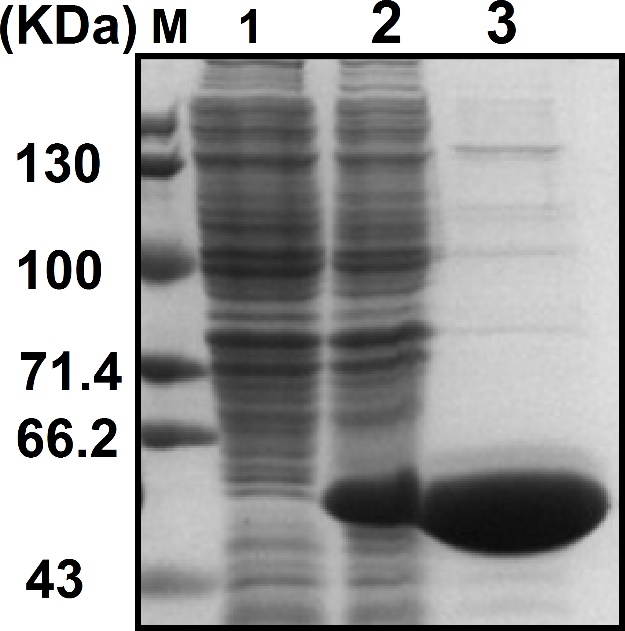


**Figure S2 Non-reducing SDS-PAGE analysis of proteins expressed in *E. coli* containing pET28a-*ohsR* plasmid.** M, broad-range protein marker; lane 1, crude extract (5 μg) without IPTG induction; lane 2, crude extract (5 μg) with induction; lane 3, loading of purified His_6_-OhsR protein.


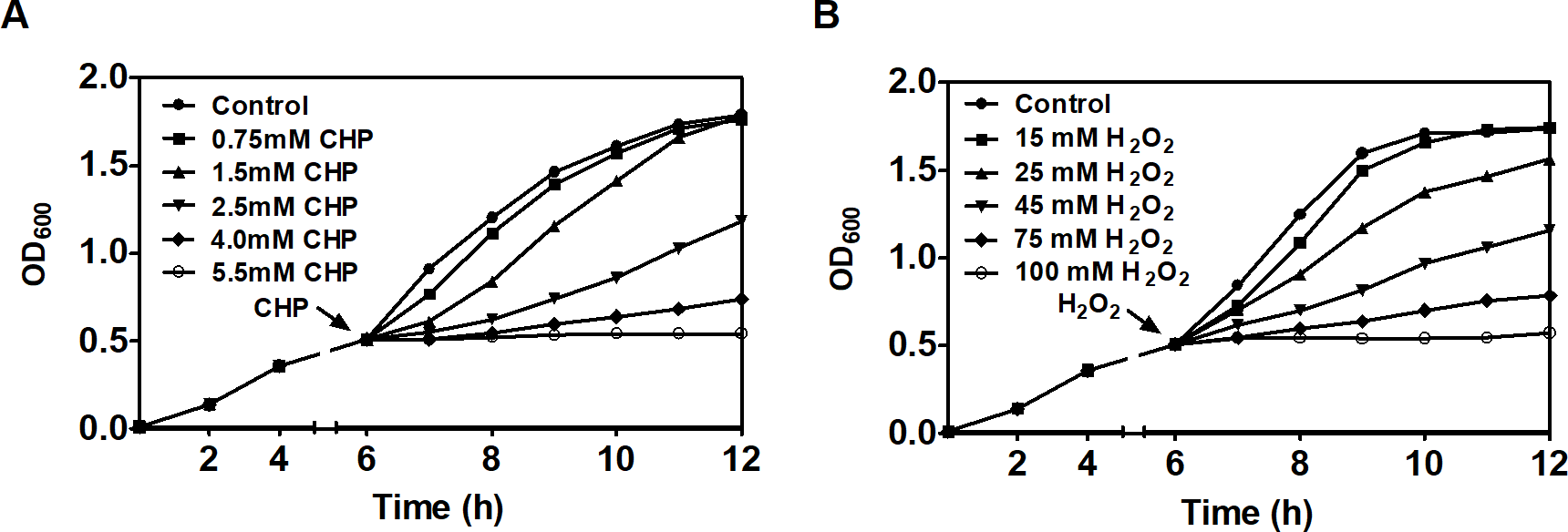


**Figure S3 Growth curves of *C. glutamicum* in response to sub-lethal concentrations of toxins.** *C. glutamicum* wild type was grown in LB medium to OD_600_ of 0.6 and exposed to different toxic agents of various concentrations. The cultures continued to be incubated for 6 h, and the OD_600_ was measured in 2 h intervals.


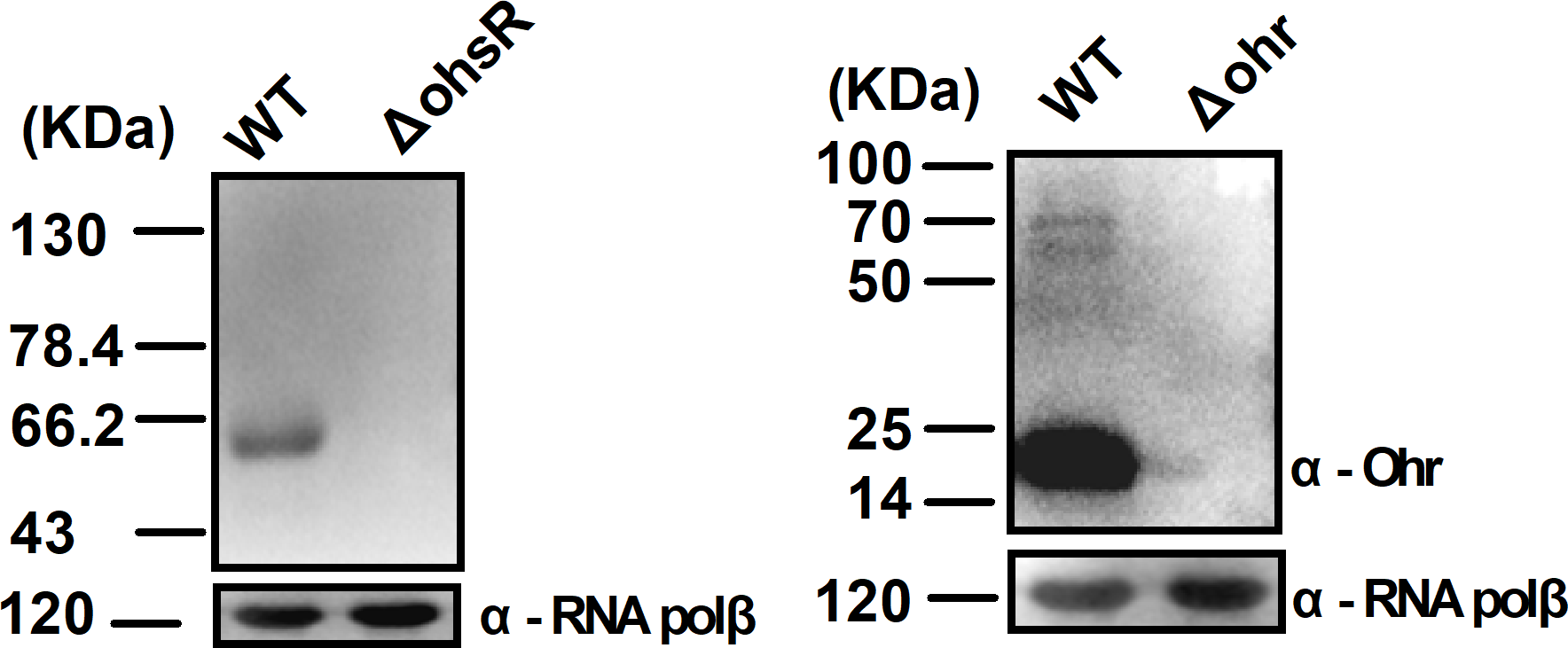


**Figure S4 The OhsR and Ohr was examined in *C. glutamicum*.** Anti-OhsR and Anti-Ohr antibodies detected a single protein with a mobility consistent with the predicted size that was absent from Δ*ohsR* and Δ*ohr* mutants. Antibodies to RNA polβ were used as a loading control.


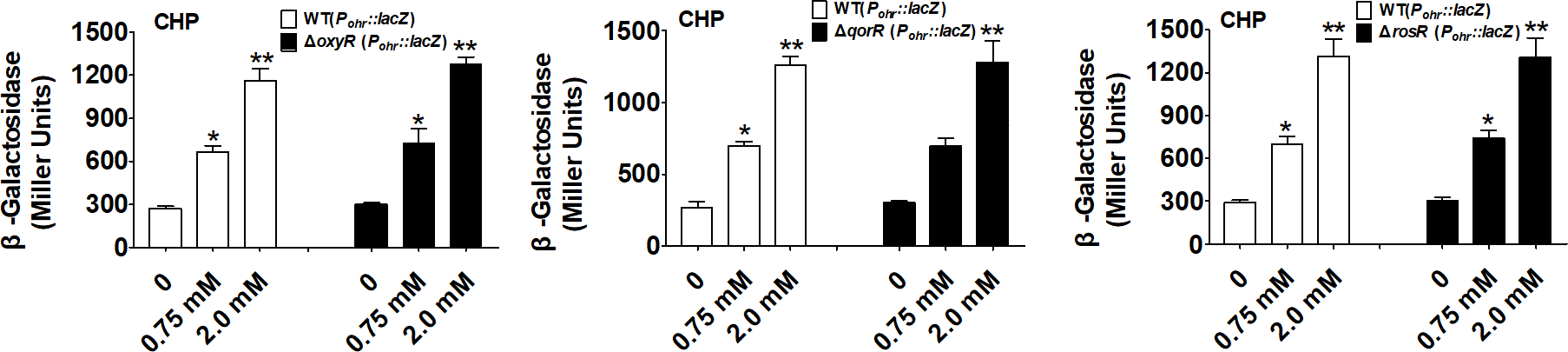


**Figure S5 Regulation of *ohr* by OxyR, QorR, and RosR.** Cells were grown in LB medium to an OD_600_ nm of 0.6. Ohr expression was analyzed 30 min after indicated CHP addition. β-Galactosidase analyses of *ohr* promoter activities by using the transcriptional *P_ohr_::lacZ* chromosomal fusion reporter expressed in indicated strains under stress conditions. **, *P* < 0.01; *: P≤0.05.


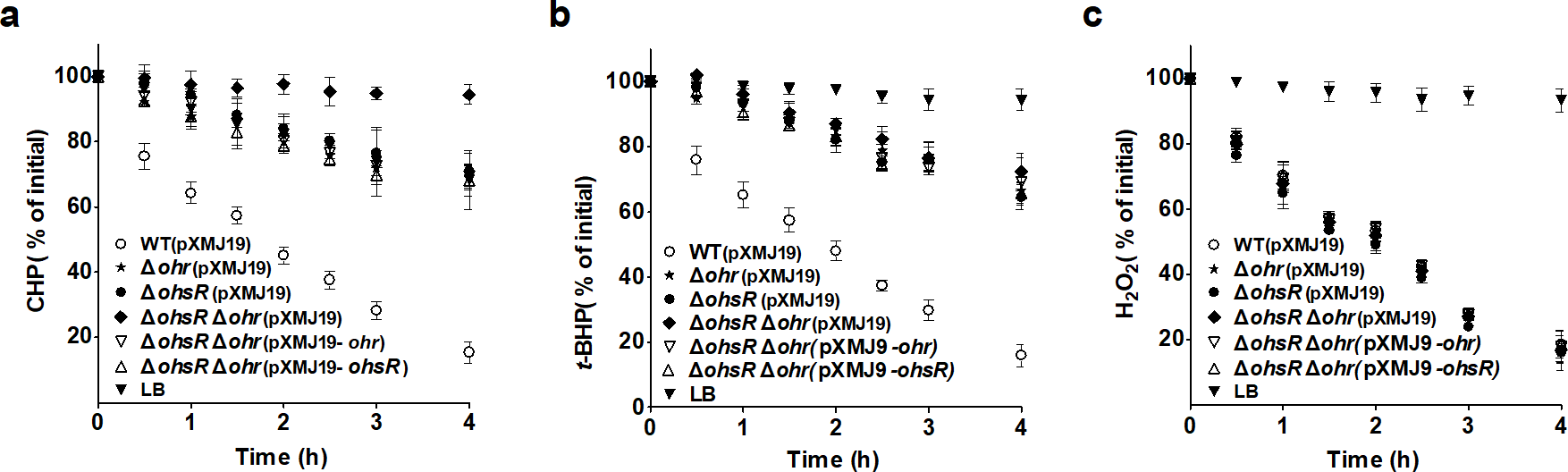


**Figure S6 Role of OhsR in scavenging peroxides.** Cultures grown in LB medium to an OD at 600 nm of 0.6 were divided and either treated with CHP (A), *t*-BHP (B), or H_2_O_2_ (C). At various time points after addition of peroxides, the level of residual peroxides remaining in the culture medium were determined with the ferrous xylenol orange (FOX) assay. Data shown are the averages of three independent experiments, and error bars indicate the SDs from three independent experiments.


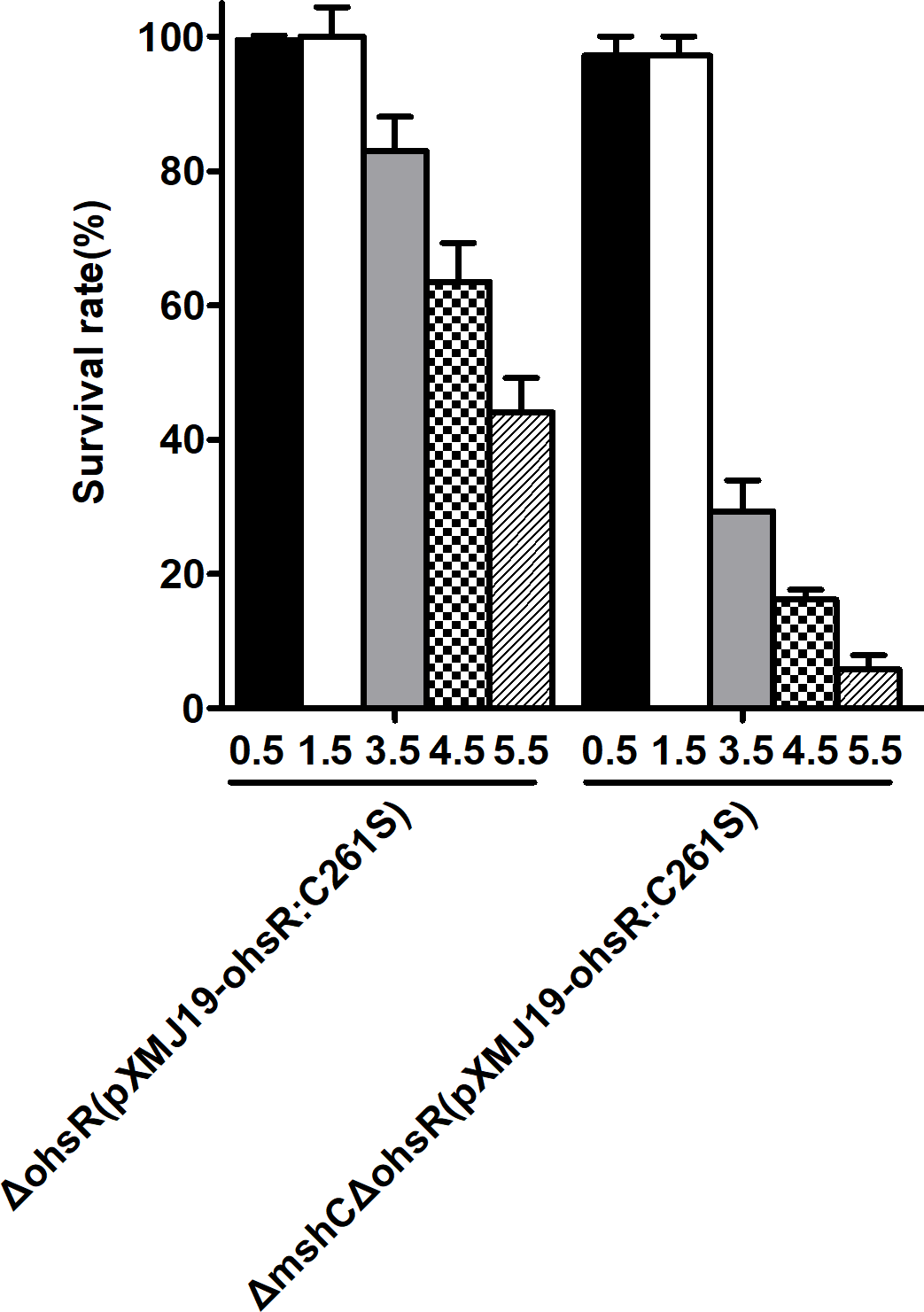


**Figure S7 Survival rates of the relevant *C. glutamicum* in response to the different concentrations of CHP.** *C. glutamicum* wild type was grown in LB medium to an OD_600_ of 0.6 and exposed to different concentrations of various CHP at 30 °C for 30 min. After treatment, the cultures were serially diluted, spreaded on LB plates and incubated at 30 °C for 36 h. Survival percentages were calculated as [(CFU ml^-1^ with stress)/(CFU ml^-1^ without stress)]×100. Mean values with standard deviations (error bars) from at least three repeats are shown.
